# Traditional Knowledge of Medicinal Plants Used by the Yao People in Lingyun County, Guangxi, China

**DOI:** 10.3390/plants15101526

**Published:** 2026-05-16

**Authors:** Wei Shen, Yuefeng Zhang, Bin Huang, Xiangtao Cen, Lingling Lv, Piyaporn Saensouk, Charun Maknoi, Khwanjai Thanakornjuk, Surapon Saensouk, Tammanoon Jitpromma

**Affiliations:** 1 Agriculture and Food Engineering College, Baise University, Baise 533000, China; bsxyshenwei@bsuc.cn (W.S.); zhangyuefeng_tj@163.com (Y.Z.); 67016660002@msu.ac.th (B.H.); nxycenxiangtao@bsuc.cn (X.C.); lvlingling207@bsuc.edu.cn (L.L.); 2 Diversity of Family Zingiberaceae and Vascular Plant for Its Applications Research Unit, Mahasarakham University, Kantharawichai District, Maha Sarakham 44150, Thailand; pcornukaempferia@yahoo.com (P.S.); jitpromma.t@gmail.com (T.J.); 3 Walai Rukhavej Botanical Research Institute, Mahasarakham University, Kantharawichai District, Maha Sarakham 44150, Thailand; 4 University Engineering Research Center for Preservation and Comprehensive Utilization of Subtropical Characteristic Agricultural Products, Baise 533000, China; 5 Department of Biology, Faculty of Science, Mahasarakham University, Kantharawichai District, Maha Sarakham 44150, Thailand; 6 Romklao Botanic Garden, The Botanical Garden Organization, Phitsanulok 65170, Thailand; charun@qsbg.org; 7 Tha Uthen Hospital, 23/23 moo.6 Nontan Thauthen, Nakhonphanom 48120, Thailand; tkjai2521@yahoo.co.th

**Keywords:** cultural importance, ethnomedicine, indigenous knowledge, Lingyun County, medicinal plants

## Abstract

Medicinal plants play a crucial role in primary healthcare among indigenous communities; however, systematic ethnomedicinal documentation of the Yao people in Lingyun County, Guangxi, remains limited. This study aimed to document the diversity, traditional uses, and cultural significance of medicinal plants used by the Yao community. Ethnobotanical data were collected through semi-structured interviews with 104 informants. A total of 172 species belonging to 135 genera and 73 families were recorded, with Fabaceae, Lamiaceae, and Asteraceae being the most represented families. Herbs were the dominant growth form, and whole plants, roots, and shoots were the most frequently used parts, typically prepared in dried form and administered orally. Fidelity Level (FL) values ranged from 6.67 to 100, with several species showing high consensus in therapeutic use. Informant Consensus Factor (ICF) values (0.778–1.000) indicated strong agreement among informants, particularly for musculoskeletal, skin, gastrointestinal, and immune-related disorders. These findings highlight the richness and consistency of ethnomedicinal knowledge among the Yao people and provide a scientific basis for future pharmacological research, conservation planning, and the sustainable use of medicinal plant resources.

## 1. Introduction

Medicinal plants have long played a fundamental role in human healthcare systems, particularly among indigenous and rural communities where traditional knowledge remains an important source of primary healthcare [1]. Across many regions of the world, plant-based remedies continue to provide accessible, affordable, and culturally appropriate treatments for a wide range of ailments [2]. Ethnobotanical studies have demonstrated that traditional medicinal knowledge represents a valuable reservoir of information for pharmacological research, biodiversity conservation, and sustainable resource management [3]. Documenting such knowledge is therefore essential, especially in regions where rapid socio-economic transformation threatens the transmission of traditional practices and cultural heritage [4].

China is rich in biological and cultural resources. The country possesses a long history of traditional medicine and extensive use of medicinal plants in healthcare systems [5]. In addition to the well-known traditional Chinese medicine (TCM), numerous ethnic minority groups maintain their own unique medical traditions that have developed through long-term interactions with local ecosystems [6]. These ethnomedicinal practices are often closely associated with cultural beliefs, local environments, and traditional lifestyles, forming an integral part of community health management and cultural identity [7].

Among the ethnic minorities in southern China, the Yao people are well known for their rich ethnomedicinal knowledge and long-standing traditions of medicinal plant use. Historically, the Yao communities have relied heavily on forest resources and surrounding natural vegetation to treat various illnesses and maintain health. Medicinal plants are used in diverse forms, including decoctions, herbal baths, poultices, and medicinal wines [8]. These traditional practices have been passed down orally from generation to generation and are often maintained by experienced elders, healers, or knowledgeable community members [9]. Despite the increasing influence of modern medicine, many Yao communities continue to depend on plant-based remedies for primary healthcare.

Guangxi Zhuang Autonomous Region, located in southern China, is characterized by complex topography, mountainous landscapes, and a subtropical climate that supports high plant diversity [10]. The region is also home to a variety of ethnic groups, including the Yao people, whose traditional knowledge of plant resources reflects long-term adaptation to the natural environment [11]. Lingyun County, situated in the northwestern part of Guangxi, possesses abundant plant diversity due to its forested mountains, favorable climate, and relatively well-preserved ecosystems. These environmental conditions provide rich medicinal plant resources that have supported the traditional healthcare practices of local communities for generations [12].

Although urbanization and socio-economic development have gradually influenced many rural areas in China, the Yao communities in Lingyun County have continued to preserve their traditional ethnomedicinal knowledge remarkably well [13]. Local residents still collect, cultivate, and use a wide variety of medicinal plants for the treatment of common illnesses and for maintaining general health [14]. Traditional knowledge related to medicinal plant identification, harvesting, preparation, and therapeutic applications remains actively practiced and transmitted within families and communities [15]. This persistence of traditional knowledge reflects the strong cultural identity of the Yao people and highlights the continuing importance of medicinal plants in their daily lives.

Despite the growing body of ethnobotanical research in China, most studies have focused on species inventories or general documentation of medicinal plant use, with relatively limited integration of quantitative indices (e.g., Fidelity Level and Informant Consensus Factor) to evaluate the consistency, reliability, and cultural significance of ethnomedicinal knowledge across communities. Furthermore, comparative understanding of how such knowledge is structured, distributed, and maintained within specific ethnic groups remains insufficient. In particular, ethnomedicinal studies focusing on the Yao people have been conducted in several regions, but systematic and quantitative documentation in Lingyun County remains lacking, representing both a geographical and analytical research gap [16].

This study addresses the following scientific questions: (1) What is the diversity and composition of medicinal plants used by the Yao people in Lingyun County? (2) How is ethnomedicinal knowledge distributed and structured among informants, as reflected by quantitative indices such as FL and ICF? and (3) Which plant species exhibit high cultural consensus and potential pharmacological relevance?

Therefore, the present study aims to document and quantitatively analyze the traditional knowledge of medicinal plants among the Yao people in Lingyun County, Guangxi, China. Specifically, this study seeks to (1) identify medicinal plant species used in local healthcare practices, (2) record their preparation methods and therapeutic applications, and (3) evaluate their cultural importance and consensus using quantitative ethnobotanical indices. By integrating qualitative documentation with quantitative analysis, this study provides a more rigorous understanding of ethnomedicinal knowledge systems and contributes to future research in ethnopharmacology, biodiversity conservation, and culturally informed healthcare systems.

## 2. Results

### 2.1. Diversity of Medicinal Plants

**Figure 1:**
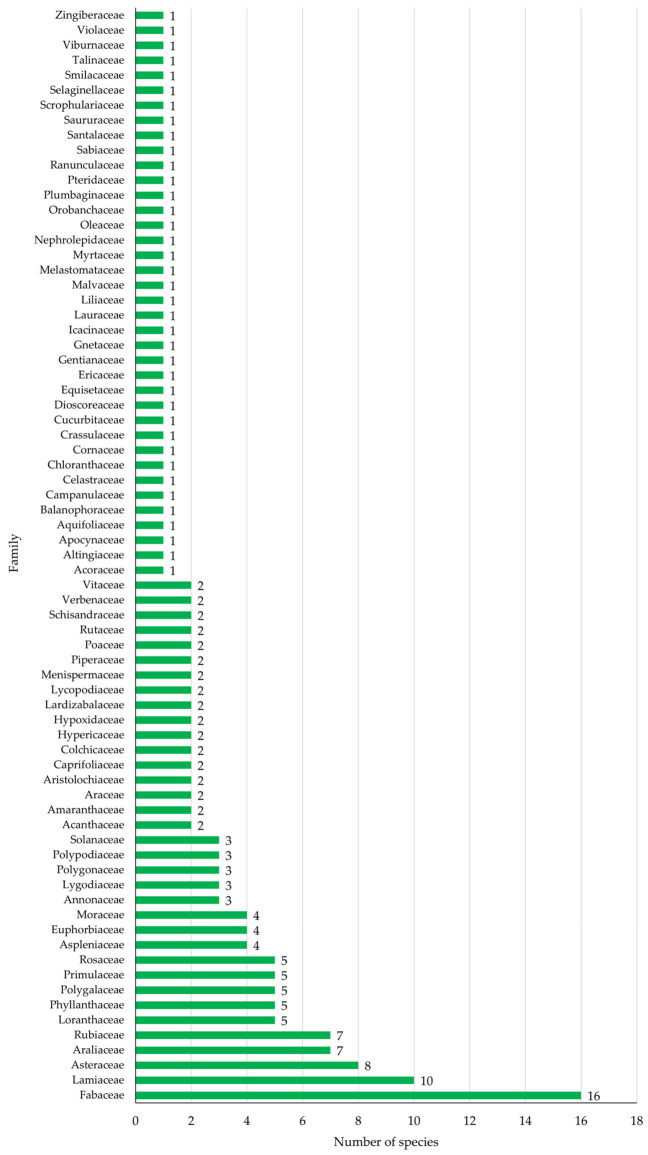
Distribution of medicinal plant diversity across plant families documented among the Yao people in Lingyun County.

**Figure 2:**
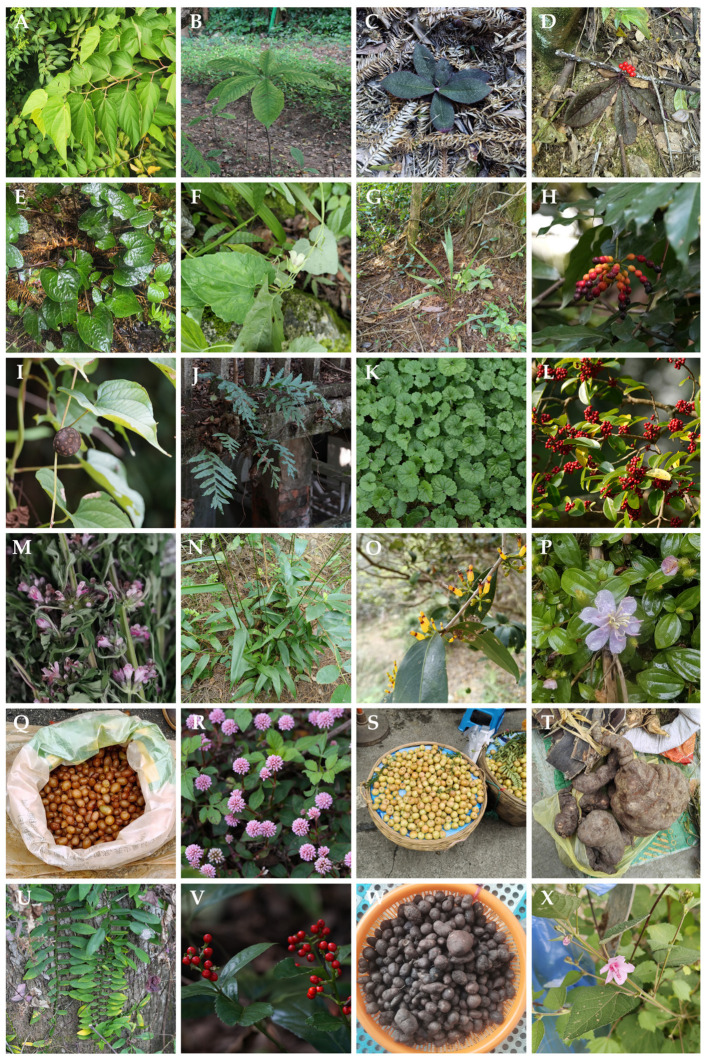
Representative examples of dried medicinal plants: (**A**) *Alangium chinense* (Lour.) Harms; (**B**) *Ardisia gigantifolia* Stapf; (**C**) *Ardisia mamillata* Hance; (**D**) *Ardisia primulifolia* Gardner & Champ.; (**E**) *Asarum caudigerum* Hance; (**F**) *Codonopsis javanica* (Blume) Hook.f. & Thomson; (**G**) *Curculigo orchioides* Gaertn.; (**H**) *Desmos chinensis* Lour.; (**I**) *Dioscorea bulbifera* L.; (**J**) *Drynaria roosii* Nakaike; (**K**) *Glechoma longituba* (Nakai) Kuprian.; (**L**) *Ilex rotunda* Thunb.; (**M**) *Leonurus japonicus* Houtt.; (**N**) *Lophatherum gracile* Brongn.; (**O**) *Macrosolen cochinchinensis* (Lour.) Tiegh.; (**P**) *Melastoma dodecandrum* Lour.; (**Q**) *Nephrolepis cordifolia* (L.) C.Presl; (**R**) *Persicaria capitata* (Buch.-Ham. ex D. Don) H. Gross; (**S**) *Phyllanthus emblica* L.; (**T**) *Pleuropterus multiflorus* (Thunb.) Turcz. ex Nakai; (**U**) *Pothos chinensis* (Raf.) Merr.; (**V**) *Sarcandra glabra* (Thunb.) Nakai; (**W**) *Tetrastigma hemsleyanum* Diels & Gilg; (**X**) *Urena lobata* L.

A total of 172 medicinal plant species belonging to 135 genera and 73 families were documented in this study among the Yao people in Lingyun County, Guangxi Zhuang Autonomous Region (Figure 1 and Figure 2). The results demonstrate a high diversity of medicinal plants traditionally used in local healthcare practices, reflecting the rich plant resources of the region and the extensive ethnomedicinal knowledge maintained within the community.

Among the recorded taxa, several plant families were particularly well represented. The family Fabaceae was the most dominant, comprising 16 species, followed by Lamiaceae with 10 species, and Asteraceae with 8 species. Other families with relatively high representation included Araliaceae and Rubiaceae, each with 7 species. In addition, Loranthaceae, Phyllanthaceae, Polygalaceae, Primulaceae, and Rosaceae each contributed 5 species to the documented medicinal flora.

Several other families showed moderate species representation, including Aspleniaceae, Euphorbiaceae, and Moraceae, each represented by 4 species. Families such as Annonaceae, Lygodiaceae, Polygonaceae, Polypodiaceae, and Solanaceae each contained 3 species. Furthermore, Acanthaceae, Amaranthaceae, Araceae, Aristolochiaceae, Caprifoliaceae, Colchicaceae, Hypericaceae, Hypoxidaceae, Lardizabalaceae, Lycopodiaceae, Menispermaceae, Piperaceae, Poaceae, Rutaceae, Schisandraceae, Verbenaceae, and Vitaceae were represented by two species each. The remaining families were represented by each single species.

### 2.2. Growth Habits of Medicinal Plants

**Figure 3:**
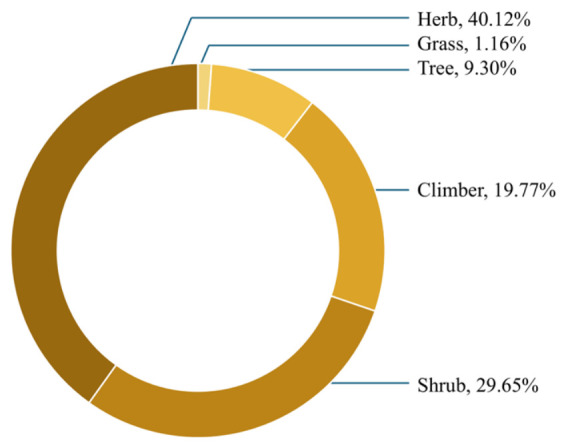
Proportion of growing habits.

Herbs were the most dominant growth habit, comprising 69 species (40.12%), followed by shrubs with 51 species (29.65%). Climbers accounted for 34 species (19.77%), while trees represented 16 species (9.30%). Grasses were the least represented group, with 2 species (1.16%) (Figure 3).

### 2.3. Used Parts of Medicinal Plants

**Figure 4:**
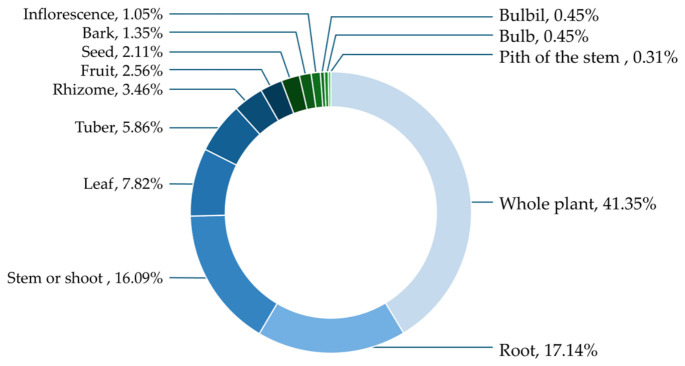
Proportion of used parts.

The analysis of plant parts used in traditional medicinal practices among the Yao people in Lingyun County, Guangxi Zhuang Autonomous Region, revealed a diverse range of plant materials utilized for therapeutic purposes. A total of 13 categories of plant parts were recorded, reflecting the flexibility and depth of traditional knowledge in the use of medicinal plant resources (Figure 4).

The whole plant was the most frequently utilized plant part, accounting for 41.35% of all recorded uses. This indicates a strong reliance on complete plant materials, particularly for herbaceous species, which are often easy to collect and process. The use of whole plants is common in traditional medicine due to the belief that the combined components may enhance therapeutic efficacy.

The second most commonly used plant parts were roots and shoots, representing 17.14% and 16.09%, respectively. Roots are widely used in traditional medicine due to their high concentration of bioactive compounds, while stems are often employed in decoctions and other preparations for treating various ailments.

Other plant parts were used less frequently. Leaves accounted for 7.82%, reflecting their accessibility and ease of preparation. Tubers and rhizomes contributed 5.86% and 3.46%, respectively, indicating their importance as underground storage organs with medicinal properties. Additionally, fruits and seeds represented 2.56% and 2.11%, respectively.

Less commonly used parts included inflorescences (1.05%), bark (1.35%), and minor categories such as bulbils (0.45%), bulbs (0.45%), and pith of the stem (0.31%). These parts were typically used in specific remedies or for particular ailments.

### 2.4. Condition of Plants Used and Routes of Administration

**Figure 5:**
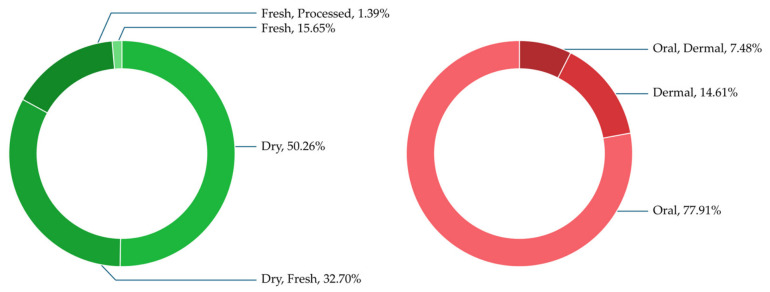
Proportion of condition of plants used and routes of administration.

Four conditions of plant materials were identified in the preparation of medicinal remedies among the Yao people in Lingyun County, Guangxi Zhuang Autonomous Region (Figure 5). Dry materials were most commonly used, accounting for 50.26% of the total, followed by the combined use of dry and fresh materials (32.70%). Fresh materials alone represented 15.65%, while fresh materials that underwent further processing accounted for 1.39%.

Three routes of administration were recorded (Figure 5). The oral route was predominant (77.91%), followed by dermal application (14.61%). The combined use of oral and dermal administration accounted for 7.48%.

### 2.5. Fidelity Level (%FL) of Medicinal Plants

Several species showed very high fidelity levels, with 100% FL, indicating that all informants consistently reported the same therapeutic use. These included *Asarum caudigerum* Hance, *A. geophilum* Hemsl., *Camphora migao* (H. W. Li) Y. Yang, Bing Liu & Zhi Yang, *Entada phaseoloides* (L.) Merr., *Equisetum ramosissimum* var. *huegelii* (Milde) Christenh. & Husby, *Eriobotrya japonica* (Thunb.) Lindl., *Flemingia prostrata* Roxb. Junior ex Roxb., *Lantana camara* L. *Melastoma dodecandrum* Lour., *Psidium guajava* L., and *Talinum paniculatum* (Jacq.) Gaertn.

High fidelity levels (≥50%) were also observed in several species, indicating strong consensus for particular treatments. For example, *Strobilanthes cusia* (Nees) Kuntze was mainly used to prevent influenza (57.14%), *Codonopsis javanica* (Blume) Hook.f. & Thomson for treating Qi deficiency and fatigue (71.43%), *Eleutherococcus nodiflorus* (Dunn) S. Y. Hu for sprains (66.67%), and *Epipremnum pinnatum* (L.) Engl. for rheumatic pain (66.67%). Similarly, *Perilla frutescens* (L.) Britton was primarily used to treat cough (60.00%), while *Striga asiatica* was commonly used for infantile diarrhea (60.00%).

Moderate fidelity levels (30–50%) were widely distributed across many species, reflecting multiple therapeutic applications. For instance, *Andrographis paniculata* (Burm.f.) Wall. ex Nees showed the highest FL for upper respiratory tract infections (45.45%), while *Acorus gramineus* Aiton was mainly used for indigestion and abdominal distension (46.15%). *Liquidambar formosana* Hance was most frequently used for bruises and swelling (42.86%), and *Achyranthes aspera* L. for bruises (37.50%). In addition, *Fissistigma oldhamii* (Hemsl.) Merr. (43.75%) and *Fissistigma polyanthum* (Hook.f. & Thomson) Merr. (38.89%) were commonly used for sprains and injuries.

Many species exhibited relatively low or distributed FL values (<30%), suggesting that they are used for multiple ailments with less consensus among informants. Examples include *Cyclea hypoglauca* (Schauer) Diels, *Dendropanax dentigerus* (Harms) Merr., *Gynura divaricata* (L.) DC., and *Hypericum japonicum* Thunb., each showing a wide range of applications across different disease categories. Detailed information on %FL values, including species-specific uses and corresponding ailments, is provided in Table S1.

### 2.6. Informant Consensus Factor (ICF) of Medicinal Plants

The Informant Consensus Factor (ICF) analysis revealed a generally high level of agreement among informants regarding the use of medicinal plants for different therapeutic categories (Table 1). The ICF values ranged from 0.778 to 1.000, indicating a strong consistency in traditional knowledge and suggesting that relatively few species are widely recognized as effective treatments for specific ailments.

The highest ICF value (1.000) was recorded for the general tonic category, although it was based on a relatively small number of use reports (N_ur_ = 10) and a single taxon (N_t_ = 1). This indicates complete agreement among informants regarding the use of this plant for general health maintenance.

Among categories with a large number of use reports, musculoskeletal disorders showed the highest ICF value (0.894), with 958 use reports and 102 taxa. This reflects a strong consensus among informants and highlights the importance of treating physical injuries and related conditions in the local community. Skin disorders also demonstrated a high level of agreement (ICF = 0.862; N_ur_ = 190, N_t_ = 27), followed by eye disorders (ICF = 0.844; N_ur_ = 33, N_t_ = 6).

Gastrointestinal disorders (ICF = 0.841; N_ur_ = 371, N_t_ = 60) and infection/immune-related disorders (ICF = 0.839; N_ur_ = 435, N_t_ = 71) were among the most frequently cited categories, both showing high consensus values. Similarly, obstetrics, gynaecology, and urinary disorders exhibited a strong level of agreement (ICF = 0.835; N_ur_ = 329, N_t_ = 55), indicating the importance of traditional medicine in addressing reproductive and urinary health issues.

Moderately high ICF values were also observed for poisoning/toxicology (0.827), respiratory disorders (0.825), and central nervous system disorders (0.818), suggesting a consistent selection of medicinal plants for these conditions. Blood disorders (ICF = 0.798) and cardiological disorders (ICF = 0.792) showed slightly lower but still substantial levels of consensus.

The lowest ICF values were recorded for reproductive disorders and cancer (both 0.778), which may reflect a broader range of plant species used for these conditions or a lower degree of shared knowledge among informants.

## 3. Discussion

The present study documents the diversity, traditional uses, and cultural significance of medicinal plants used by the Yao people in Lingyun County, with a total of 172 species belonging to 135 genera and 73 families recorded. These findings reflect not only the rich plant resources of the region but also the depth of ethnomedicinal knowledge embedded within the community. Such diversity is closely associated with ecologically heterogeneous mountainous environments and long-term human–environment interactions, which shape both plant availability and the development, transmission, and adaptation of traditional knowledge systems [17,18].

The dominance of families such as Fabaceae, Lamiaceae, and Asteraceae observed in this study is consistent with widely reported ethnobotanical patterns [19,20]. These families are known for their ecological adaptability and their richness in bioactive secondary metabolites, including alkaloids, terpenoids, and phenolic compounds [21,22]. Their prominence across diverse traditional medical systems suggests a non-random selection of plant taxa, reflecting a convergence between traditional knowledge and phytochemical potential [23].

The predominance of herbaceous species (40.12%) reflects a consistent ethnobotanical pattern observed across diverse traditional medical systems, where herbs are preferentially selected due to their high accessibility, rapid life cycles, and ease of harvest without specialized tools [24]. Beyond practical convenience, this preference also reflects adaptive knowledge of ecological resilience, as herbaceous plants generally exhibit faster regeneration and lower extraction cost to local ecosystems [25]. In contrast, the lower representation of trees suggests more than simple scarcity; it also reflects deliberate harvesting avoidance driven by both ecological constraints and culturally informed sustainability practices. Trees typically require higher labor input for collection, exhibit slower recovery rates, and often hold ecological or symbolic value that discourages intensive use [26]. Overall, these patterns demonstrate that plant selection in Yao ethnomedicine is not solely resource-driven but is embedded within a complex system of ecological awareness, experiential knowledge, and implicit resource management strategies that collectively contribute to the long-term sustainability of medicinal plant use [27].

The analysis of plant parts used reveals a strong reliance on whole plants (41.35%) and roots (17.14%), which raises important sustainability considerations. While the use of whole plants may be associated with perceived synergistic effects among plant components, harvesting entire individuals—particularly herbaceous taxa—can negatively affect population dynamics [28]. Root harvesting is especially critical, as it often leads to plant mortality and reduced regeneration capacity [29].

In addition to these ecological concerns, harvesting practices are often mediated by local customary norms and traditional ecological knowledge. Although formal regulatory systems were not explicitly documented in this study, informants indicated that plant collection is influenced by seasonal availability, selective harvesting, and culturally embedded practices that help avoid excessive exploitation, particularly for less abundant species. Such informal management systems, including selective collection and awareness of habitat conditions, may contribute to the sustainability of medicinal plant resources. However, increasing market integration and socio-economic change may weaken these traditional controls, highlighting the need to integrate local knowledge into broader conservation strategies [30,31].

The predominance of dried plant materials and oral administration reflects not only practical adaptations to storage and seasonality but also a scientifically consistent strategy for preserving phytochemical integrity. Drying is widely recognized as a critical post-harvest process that reduces enzymatic activity and microbial proliferation by lowering water activity, thereby extending shelf life and stabilizing medicinal plant materials. Importantly, recent evidence demonstrates that drying conditions directly influence the stability of key bioactive compounds, including phenolics, flavonoids, and volatile constituents, through temperature-dependent processes such as oxidation, enzymatic inactivation, and volatilization. Although thermal exposure may induce partial degradation of heat-sensitive metabolites, controlled drying can also enhance compound stability by shortening drying time and reducing enzymatic breakdown, thereby optimizing the retention of antioxidant-related phytochemicals [32].

The dominance of oral administration reflects the widespread use of decoctions and infusions as primary extraction methods in traditional medicine systems. These preparations efficiently extract polar and semi-polar bioactive compounds, such as polysaccharides, glycosides, phenolic acids, and flavonoids, which are commonly associated with therapeutic effects. Prolonged heating during decoction enhances cellular breakdown and metabolite release, but may also reduce thermolabile or volatile constituents. Overall, this preference represents a practical and empirically optimized strategy that balances extraction efficiency, compound solubility, and physiological absorption [33].

The patterns observed in FL and ICF collectively reveal a structured yet dynamically adaptive ethnomedicinal knowledge system among the Yao community in Lingyun County [34]. Beyond descriptive ethnobotanical interpretation, these patterns can be theoretically situated within the framework of Cultural Consensus Theory (CCT) as proposed by Romney et al., 1986 [35], which conceptualizes cultural knowledge as a shared cognitive model inferred from the degree of agreement among informants.

From this perspective, high-FL species with complete or near-complete informant agreement (e.g., *Equisetum ramosissimum*, *Eriobotrya japonica*, *Psidium guajava*) represent domains of high cultural consensus and high shared competence. In CCT terms, such convergence suggests that informants operate with a strongly overlapping cultural model regarding the medicinal function of these taxa, indicating both high “truth consensus” and high cultural salience. These species can therefore be interpreted as elements of a cognitively stable ethnopharmacological domain, where knowledge has been repeatedly validated, socially reinforced, and transmitted with minimal variation across individuals [36,37].

In contrast, species exhibiting moderate to low FL values reflect lower agreement in cultural knowledge domains, which within the CCT framework may indicate either (i) reduced shared competence among informants or (ii) the presence of multiple coexisting cultural models regarding plant use [38]. Rather than representing knowledge weakness, this variation highlights the pluralistic nature of ethnomedical cognition, where different informants may rely on distinct experiential, ecological, or heuristic frameworks to assign therapeutic functions to the same taxa [39].

At a broader structural level, the generally high ICF values (0.778–1.000) further reinforce the presence of a coherent shared cultural model at the system level [40]. In CCT terms, high ICF reflects strong agreement in the “cultural domain structure,” where informants consistently converge on a limited set of taxa for specific illness categories [41]. This indicates that despite variation at the species-use level, there exists a high degree of shared semantic organization of illness categories, particularly for musculoskeletal, gastrointestinal, and infection-related disorders. These categories can thus be interpreted as high-probability domains within the collective cultural model of health and illness [42].

The highest consensus was observed in the general tonic category (ICF = 1.000; N_ur_ = 10, N_t_ = 1), indicating complete agreement among informants. However, this result is based on a very limited number of use reports and a single taxon, and is therefore not directly comparable in robustness to higher-representation categories such as musculoskeletal disorders.

The integration of CCT with ethnobotanical indices also allows for a more refined interpretation of knowledge distribution. High consensus disease categories (high ICF) correspond to domains where cultural models are highly stabilized, likely due to a combination of high disease prevalence, frequent experiential feedback, and repeated intergenerational validation [43]. This results in strong cognitive alignment among informants, consistent with the prediction of CCT that high agreement domains reflect high cultural competence and well-defined shared knowledge structures [44].

Conversely, lower ICF values in categories such as reproductive disorders and cancer suggest lower cultural agreement and higher cognitive variability, which may reflect either limited experiential exposure, reduced treatment visibility, or the coexistence of multiple interpretive models of illness causation. Within the CCT framework, such domains are characterized by weaker signal-to-noise ratios in cultural knowledge transmission, leading to more diffuse agreement patterns and less structured consensus [45].

When interpreted jointly, FL, ICF, and CCT provide a multi-scalar understanding of ethnomedicinal knowledge. FL captures item-level cultural consensus (specific plant–use agreement), ICF reflects domain-level consensus (disease category structure), and CCT provides the overarching theoretical lens that explains both as manifestations of a shared cognitive system with varying degrees of internal consistency. This multi-layered structure reveals that ethnomedicinal knowledge among the Yao is not random or purely experiential, but instead reflects a cognitively organized cultural system characterized by both high internal agreement in core domains and adaptive variability in peripheral ones [46].

An additional dimension emerging from this study is the overlap between medicinal and dietary plant use. Several documented species, including *Phyllanthus emblica* and members of the Zingiberaceae, are commonly consumed as part of the local diet while also serving therapeutic functions. This reflects the well-established food–medicine continuum, in which the boundary between food and medicine is fluid and dietary practices contribute directly to health maintenance and disease prevention [47]. Such dual-use species are increasingly recognized in functional food and nutraceutical research due to their combined cultural acceptance and bioactive potential. The documentation of these species within the Yao community therefore provides valuable insights for future interdisciplinary research linking ethnobotany, nutrition, and pharmacology [48,49].

Comparative analysis with other ethnobotanical studies in China and neighboring regions reveals both convergence and local specificity. While dominant plant families and general usage patterns are broadly consistent, species composition and therapeutic applications vary across regions. These differences are shaped by micro-ecological variation, cultural identity, and historical factors, emphasizing the importance of localized ethnobotanical research [8,18,41,48,49].

Despite the richness of the documented knowledge, its long-term persistence remains uncertain in the context of ongoing socio-economic transformation, modernization, and intergenerational discontinuity. Although the relatively high consensus observed in this study indicates that ethnomedicinal knowledge is still actively practiced and socially reinforced, its continuity cannot be assumed without deliberate intervention [50,51]. Future research should therefore move beyond documentation toward (i) longitudinal monitoring of knowledge transmission across generations, (ii) comparative studies between age groups and villages to detect erosion or transformation of knowledge systems, and (iii) integration of ethnobotanical datasets with ecological and conservation assessments to identify vulnerable knowledge–plant linkages. In parallel, applied research should prioritize pharmacological validation of high-consensus species and evaluate their potential incorporation into community-based healthcare and sustainable cultivation systems, ensuring both cultural continuity and resource resilience [52].

In conclusion, this study demonstrates that Yao ethnomedicinal knowledge in Lingyun County is not only diverse but also structurally coherent and functionally adaptive. Beyond contributing a systematic inventory of medicinal plant use, the findings provide a scientific foundation for future interdisciplinary research linking ethnobotany, conservation biology, and natural product development. This includes the identification of candidate species for bioactivity screening, the exploration of food–medicine dual-use plants for functional food applications, and the development of community-informed conservation strategies that support both biodiversity preservation and cultural sustainability.

## 4. Materials and Methods

### 4.1. Study Area

**Figure 6:**
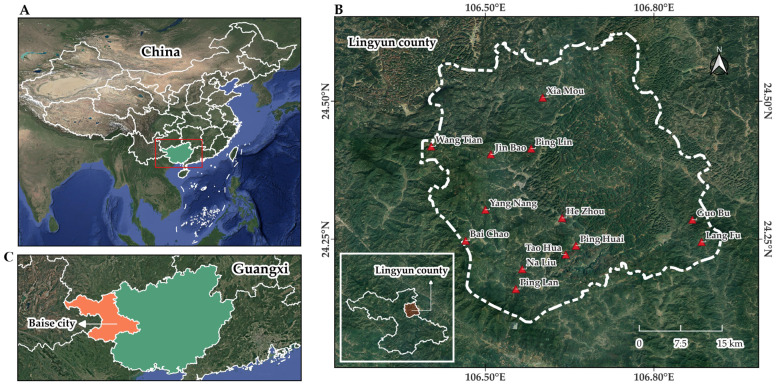
Map of the study area: The top-left map (**A**) highlights Guangxi Zhuang Autonomous Region in green within China. The red box indicates its location within China. The bottom-left map (**C**) shows Baise City in orange within Guangxi. The right map (**B**) indicates the study location in Lingyun County (map created with “QGIS” program ver. 3.34 [54], geographic system ID: WGS 84, EPSG 4326).

Lingyun County is located in the northwestern part of the Guangxi Zhuang Autonomous Region in southern China and is administratively under Baise City (Figure 6). Geographically, the county lies in a transitional zone between the Yungui Plateau and the hilly mountainous regions of Guangxi, extending between 106°23′–106°55′ E and 24°06′–24°37′ N. Lingyun County covers an area of approximately 2048 km^2^, with mountainous terrain accounting for about 93.32% of the total land area. The county has a population of approximately 188,000 inhabitants [53].

The topography of Lingyun County is characterized by higher elevations in the northwest and relatively lower terrain in the southeast, with elevations ranging from approximately 191 m to more than 2000 m above sea level. The landscape mainly consists of clastic rock formations in the western areas and carbonate rock formations in the eastern areas, resulting in extensive karst landforms, steep slopes, and deep valleys. Several rivers belonging to the Youjiang River and Hongshui River systems flow through the county, supporting local hydrological processes and agricultural activities in valley areas [55]. Lingyun County experiences a humid subtropical monsoon climate (Köppen Cfa), with a mean annual temperature of approximately 20.7 °C and an average annual precipitation of about 1729 mm. Nearly 80% of the annual rainfall occurs during the monsoon season from May to September. The county maintains a forest coverage rate of approximately 71%, providing favorable environmental conditions for high plant diversity and the availability of medicinal plant resources [56].

The county is ethnically diverse. According to the seventh national population census (2020), Lingyun County is home to 25 recognized ethnic groups. Han Chinese constitute the largest proportion of the population (44.08%), while ethnic minorities account for 55.92%, including the Zhuang people (31.23%) and the Yao people (24.69%) [57]. Among these groups, the Yao people possess a rich cultural heritage and maintain traditional knowledge related to the use of plant resources.

Resource use is often guided by seasonal calendars, ecological indicators, and customary norms that help regulate harvesting activities and promote sustainable utilization of plant resources. Although modernization, improved transportation, and increasing market integration have gradually influenced local lifestyles, the use of medicinal plants remains culturally significant among the Yao people. Mandarin Chinese and local dialects are commonly used in daily communication, while the Yao language is still spoken in some villages, particularly among older community members. The rich plant diversity and well-preserved traditional knowledge in Lingyun County make the area an important site for ethnobotanical research on medicinal plant use.

### 4.2. Medicinal Plant Collection and Identification

All medicinal plant species reported by informants were collected through guided field walks conducted in villages, as well as visits to local markets and traditional herbal medicine shops. During collection, specimens were photographed in their natural habitats and subsequently processed into herbarium vouchers following standard preservation techniques. Each specimen was assigned a unique voucher number, and all voucher numbers are provided in Table S1. The prepared specimens were deposited at the Baise University, located in Baise city, Guangxi Zhuang Autonomous Region, China, to ensure proper curation and long-term storage.

**Figure 7:**
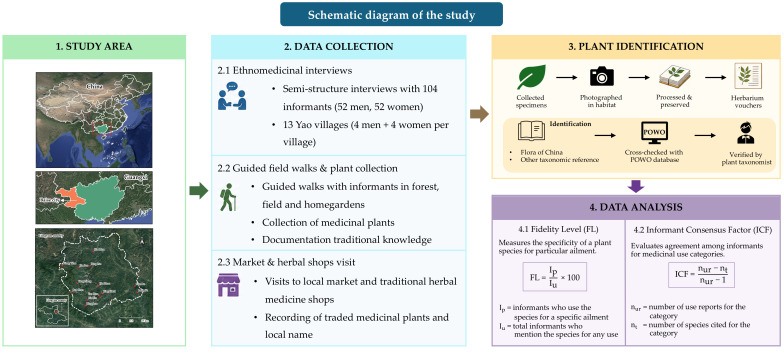
Schematic diagram of the study.

Identification of plant species was initially conducted using regional floras, particularly Flora of China, along with other relevant taxonomic references. To ensure accuracy, the scientific names and family assignments were further cross-checked and validated using the Plants of the World Online (POWO) database [58]. In addition, all identifications were verified by an experienced plant taxonomist (Surapon Saensouk), the corresponding author of this study (Figure 7).

### 4.3. Ethnomedicinal Interviews and Informant Selection

In 2025, ethnomedicinal fieldwork was carried out across 13 villages primarily inhabited by the Yao ethnic group. The study employed qualitative ethnobotanical approaches, including semi-structured interviews, participant observation, and guided field walks with knowledgeable local informants.

Semi-structured interviews were conducted using a flexible interview guide consisting of open-ended questions covering local plant names, parts used, preparation methods, therapeutic applications, and modes of administration. This approach allowed informants to freely elaborate on their knowledge while ensuring consistency across interviews.

A total of 104 informants (52 men and 52 women), aged between 21 and 75 years (Table 2), were selected using purposive [59] and snowball sampling techniques [60]. Purposive sampling was initially applied to identify key informants, including traditional healers, village elders, and experienced plant users recognized within the community for their ethnomedicinal knowledge. Subsequently, snowball sampling was employed by asking initial informants to recommend other knowledgeable individuals within their social networks. In each village, four male and four female participants were selected to ensure gender-balanced representation. Informants were selected based on specific inclusion criteria to ensure relevance and reliability of the data: (1) long-term residence in the study area, defined as individuals who were born and raised in the area or had continuously lived there for at least 10 years; (2) active use of medicinal plants for healthcare, either for self-treatment or within the household; and (3) recognition by community members as knowledgeable plant users or practitioners.

Interviews were conducted in either Mandarin Chinese dialect, covering topics such as vernacular plant names, plant parts used, preparation and administration techniques, collection locations, and the cultural or symbolic importance of each species. Additional ethnobotanical information was documented through direct observation and hands-on participation during plant collection activities.

Prior to the start of data collection, the study objectives and procedures were clearly communicated to all participants, and written or verbal informed consent was obtained in accordance with the International Society of Ethnobiology (ISE) Code of Ethics [61] and the Nagoya Protocol on Access and Benefit-Sharing [62]. Participants were informed of their rights, including voluntary participation and the freedom to withdraw at any time. While the study did not collect sensitive personal data and therefore did not require formal institutional ethical approval, all activities adhered to internationally recognized ethical standards for ethnobotanical research, ensuring respect, transparency, and reciprocity toward the participating communities.

### 4.4. Data Analysis

#### 4.4.1. Fidelity Level (%FL)

The Fidelity Level (FL) was used to quantify the extent to which a specific plant species is linked to a particular medicinal application. This index reflects the proportion of informants who associate a species with the same therapeutic use, providing insights into the cultural significance and perceived reliability of traditional remedies. FL was calculated following Friedman et al. [63] using the formula:

(1) $$\mathrm{FL}\;=\;\frac{I_{p}}{I_{u}}\;\times \;100$$

where I_p_ represents the number of informants reporting the use of a species for a specific ailment, and I_u_ is the total number of informants mentioning the species for any medicinal purpose. Higher FL values indicate that the majority of informants consistently link the species to a single, well-defined use, demonstrating strong cultural agreement and specificity in ethnomedicinal knowledge.

#### 4.4.2. Informant Consensus Factor (ICF)

The Informant Consensus Factor (ICF) was applied to evaluate the level of agreement among informants regarding the use of medicinal plants for particular medicinal purposes. This metric helps identify which ailment categories have strong shared knowledge and consistent use of specific plant species within the community. Fic was calculated following the method of Heinrich et al. [64] using the formula:

(2) $$\mathrm{ICF}=\frac{n_{\mathrm{ur}}-n_{t}}{n_{\mathrm{ur}}-1}$$

where n_ur_ is the total number of use reports for a given medicinal category, and n_t_ is the number of species cited for that category. ICF values range from 0 to 1, with values closer to 1 indicating a high degree of consensus among informants. High ICF values suggest that certain ailments are widely recognized and that particular plant remedies are consistently preferred and trusted by the local community.

## 5. Conclusions

This study provides a comprehensive documentation of 172 medicinal plant species used by the Yao people in Lingyun County, Guangxi Zhuang Autonomous Region, reflecting both the rich plant diversity and extensive traditional knowledge in the region. Herbs were the predominant growth habit, and the whole plant, roots, and shoots were most commonly used, indicating practical preparation preferences in local medicine.

Fidelity level (%FL) and Informant Consensus Factor (ICF) analyses revealed strong agreement among informants for specific therapeutic uses, particularly for musculoskeletal, skin, gastrointestinal, and immune-related disorders. These findings highlight species with high medicinal significance, which may serve as candidates for further pharmacological and conservation studies.

Overall, this work emphasizes the importance of preserving ethnomedicinal knowledge and sustainable use of plant resources, providing a valuable baseline for both scientific research and cultural heritage conservation.

## Figures and Tables

**Table 1:** Informant Consensus Factor (ICF) values for different therapeutic categories of medicinal plants used by the Yao people in Lingyun County.

Therapeutic Categories	N_ur_	N_t_	ICF
General Tonic	10	1	1.000
Musculoskeletal Disorders	958	102	0.894
Skin Disorders	190	27	0.862
Eye Disorders	33	6	0.844
Gastrointestinal Disorders	371	60	0.841
Infection/Immune Disorders	435	71	0.839
Obstetrics, Gynaecology and Urinary Disorders	329	55	0.835
Poisoning/Toxicology	82	15	0.827
Respiratory Disorders	281	50	0.825
Central Nervous System Disorders	56	11	0.818
Blood Disorders	100	21	0.798
Cardiological Disorders	25	6	0.792
Reproductive Disorders	19	5	0.778
Cancer	10	3	0.778

**Table 2:** Demographic characteristics of the surveyed in Lingyun County.

Name of Village	GPS Coordinates		Gender		Ethnicity
	Latitude (N, S)	Longitude (E, W)	Male	Female	
Bai Chao	24°14′50.8684″	106°27′53.7401″	4	4	Indigo-dyed Yao
Guo Bu	24°17′07.1357″	106°52′06.0339″	4	4	Indigo-dyed Yao
He Zhou	24°17′18.5071″	106°38′12.0107″	4	4	Indigo-dyed Yao
Jin Bao	24°24′13.7642″	106°30′35.0621″	4	4	Indigo-dyed Yao
Lang Fu	24°14′42.1523″	106°53′04.9932″	4	4	Indigo-dyed Yao
Na Liu	24°11′45.3460″	106°33′56.3983″	4	4	Indigo-dyed Yao
Ping Huai	24°14′17.7814″	106°39′40.0730″	4	4	Indigo-dyed Yao
Ping Lan	24°09′35.3969″	106°33′15.1095″	4	4	Indigo-dyed Yao
Ping Lin	24°24′51.1780″	106°34′53.5809″	4	4	Indigo-dyed Yao
Tao Hua	24°13′19.8954″	106°38′35.3588″	4	4	Basket-carrying Yao
Wang Tian	24°25′05.0084″	106°24′13.0533″	4	4	Indigo-dyed Yao
Xia Mou	24°30′23.5896″	106°36′07.5648″	4	4	Indigo-dyed Yao
Yang Nang	24°18′11.8925″	106°30′00.4068″	4	4	Basket-carrying Yao

## Data Availability

The original contributions of this study are provided within the article, and any additional inquiries may be addressed to the corresponding author.

## Supplementary Materials

The following supporting information can be downloaded at: https://www.mdpi.com/article/10.3390/plants15101526/s1, Table S1: Comprehensive list of medicinal plants used by the Yao people in Lingyun County, Guangxi, China, including family, scientific name, growth habit, vernacular name, voucher number (VN), fidelity level (%FL), used parts (UP), condition of plant materials (CoP), routes of administration (RoA), preparation methods, therapeutic applications, and categorized therapeutic uses.
