# Supplementary material for: Traditional Knowledge of Medicinal Plants Used by the Yao People in Lingyun County, Guangxi, China
Source: Plants (Basel). 2026 May 16;15(10):1526. doi: 10.3390/plants15101526 (PMC13210878; doi:10.3390/plants15101526)
Supplement: Supplementary file 1 [file plants-15-01526-s001.zip › plants-4258421-supplementary.pdf]

Supplementary

Traditional Knowledge of Medicinal Plants Among the Yao People in Lingyun County, Guangxi, China

Table S1. Comprehensive list of medicinal plants used by the Yao people in Lingyun County, Guangxi, China, including family, scientific name, growth habit, vernacular name, voucher number (VN), fidelity level (%FL), used parts (UP), condition of plant materials (CoP), routes of administration (RoA), preparation methods, therapeutic applications, and categorized therapeutic uses.

Family	Scientific name	Growth habit	Vernacular name	VN	FL	UP	CoP	RoA	Preparation	Application	Therapeutic Categories
Acanthaceae	*Andrographis paniculata* (Burm.f.) Wall. ex Nees	Herb	chuān xīn lián	WS-MD0001	18.18	Wp	Dry	Oral	Boiled in water and the filtrate is taken orally.	Used to treat colds and fever.	Infection/Immune Disorders
					36.36	Wp	Dry	Oral	Boiled in water and the filtrate is taken orally.	Used to treat tonsillitis.	Respiratory Disorders
					45.45	Wp	Dry	Oral	Boiled in water and the filtrate is taken orally.	Used to treat upper respiratory tract infections.	Respiratory Disorders
	Strobilanthes cusia (Nees) Kuntze	Herb	bǎn lán,bǎn lán gēn	WS-MD0127	57.14	Wp	Dry, Fresh	Oral	Boiled in water and the filtrate is taken orally.	Used to prevent influenza.	Infection/Immune Disorders
					42.86	Wp	Dry, Fresh	Oral	Boiled in water and the filtrate is taken orally.	Used to treat acute enteritis and pharyngitis.	Gastrointestinal Disorders
Acoraceae	*Acorus gramineus* Aiton	Herb	shí chāng pú	WS-MD0012	30.77	Rz	Dry, Fresh	Oral	Boiled in water and the filtrate is taken orally.	Used to treat wind-cold-dampness syndrome.	Respiratory Disorders
					46.15	Rz	Dry, Fresh	Oral	Boiled in water and the filtrate is taken orally.	Used to treat indigestion and abdominal distension.	Gastrointestinal Disorders

					23.08	Rz	Dry, Fresh	Oral	Boiled in water and the filtrate is taken orally.	Used to treat chest tightness.	Cardiological Disorders
Altingiaceae	*Liquidambar formosana* Hance	Tree	fēng xiāng,lù lù tōng	WS-MD0118	35.71	Ft, Lv, Rt	Dry	Oral	Boiled in water and the filtrate is taken orally.	Used to treat rheumatic pain.	Musculoskeletal Disorders
					21.43	Ft, Lv, Rt	Dry	Oral	Boiled in water and the filtrate is taken orally.	Used to treat hemiplegia due to stroke.	Central Nervous System Disorders
					42.86	Lv	Fresh	Dermal	For external use, pounded and applied to the affected area.	Used to treat bruises and swelling from falls.	Musculoskeletal Disorders
Amaranthaceae	*Achyranthes aspera* L.	Herb	tǔ niú xī,niú xī fēng,dǎo gōu cǎo	WS-MD0011	12.50	Wp	Dry, Fresh	Oral	Boiled in water and the filtrate is taken orally.	Used to treat colds and fever.	Infection/Immune Disorders
					25.00	Wp	Dry, Fresh	Oral	Boiled in water and the filtrate is taken orally.	Used to treat tonsillitis.	Respiratory Disorders
					25.00	Rt	Dry, Fresh	Oral	Taken orally as a decoction or alcohol infusion	Used to treat rheumatic bone pain.	Musculoskeletal Disorders
					37.50	Rt	Dry, Fresh	Oral	Taken orally as a decoction or alcohol infusion	Used to treat bruises.	Musculoskeletal Disorders
	Celosia argentea L.	Herb	qīng xiāng, yě jī guān huā	WS-MD0049	42.86	Sd	Dry	Oral	Boiled in water and the filtrate is taken orally.	Used to treat red, swollen, and painful eyes and acute conjunctivitis.	Eye Disorders
					35.71	Sd	Dry	Oral	Boiled in water and the filtrate is taken orally.	Used to treat hypertension.	Blood Disorders
					21.43	Sd	Dry	Oral	Boiled in water and the filtrate is taken orally.	Used to treat dizziness.	Central Nervous System Disorders
Annonaceae	*Desmos chinensis* Lour.	Shrub	jī zhǎo fēng, jiǎ yīng zhǎo	WS-MD0060	41.67	Bk	Dry	Oral	Boiled in water and the filtrate is taken orally.	Used to treat rheumatic bone pain.	Musculoskeletal Disorders
					58.33	Bk	Dry	Oral	Boiled in water and the filtrate is taken orally.	Used to treat traumatic injuries.	Musculoskeletal Disorders

	Fissistigma oldhamii (Hemsl.) Merr.	Shrub	tiě zuàn, guā fù mù	WS-MD0088	25.00	Rt	Dry	Oral	Taken orally as a decoction or alcohol infusion.	Used to treat rheumatoid arthritis.	Musculoskeletal Disorders
					31.25	Rt	Dry	Oral	Taken orally as a decoction or alcohol infusion.	Used to treat lower back and leg pain.	Musculoskeletal Disorders
					43.75	Rt	Dry	Oral	Taken orally as a decoction or alcohol infusion.	Used to treat sprains and bruises.	Musculoskeletal Disorders
	Fissistigma polyanthum (Hook.f. & Thomson) Merr.	Shrub	hēi fēng téng, niú ěr fēng	WS-MD0089	38.89	Rt, St	Dry	Oral	Taken orally as a decoction or alcohol infusion.	Used to treat sprains and bruises.	Musculoskeletal Disorders
					22.22	Rt, St	Dry	Oral	Taken orally as a decoction or alcohol infusion.	Used to treat rheumatoid arthritis.	Musculoskeletal Disorders
					27.78	Rt, St	Dry	Oral	Boiled in water and the filtrate is taken orally.	Used to treat colds.	Infection/Immune Disorders
					11.11	Rt, St	Dry	Oral	Boiled in water and the filtrate is taken orally.	Used to treat menstrual disorders.	Obstetrics, Gynaecology and Urinary Disorders
Apocynaceae	*Trachelospermum jasminoides* (Lindl.) Lem.	Climber	pá qiáng fēng, luò shí, ruǎn jīn téng	WS-MD0136	14.29	Lv, St	Dry, Fresh	Oral	Boiled in water and the filtrate is taken orally.	Used to treat rheumatic pain.	Musculoskeletal Disorders
					35.71	Lv, St	Dry, Fresh	Oral	Boiled in water and the filtrate is taken orally.	Used to treat traumatic injuries.	Musculoskeletal Disorders
					50.00	Lv, St	Dry, Fresh	Oral	Boiled in water and the filtrate is taken orally.	Used to treat postpartum lochia retention.	Obstetrics, Gynaecology and Urinary Disorders
Aquifoliaceae	*Ilex rotunda* Thunb.	Tree	jiù bì yìng, jiǔ céng pí	WS-MD0107	21.43	Bk	Dry	Oral	Boiled in water and the filtrate is taken orally.	Used to treat high fever due to colds and sore throat.	Infection/Immune Disorders
					35.71	Bk	Dry	Oral	Boiled in water and the filtrate is taken orally.	Used to treat nephritis edema.	Obstetrics, Gynaecology and Urinary Disorders

					42.86	Bk	Dry, Fresh	Oral, Dermal	Boiled in water and the filtrate is taken orally. For external use, decoction for washing.	Used to treat fractures.	Musculoskeletal Disorders
Araceae	*Epipremnum pinnatum* (L.) Engl.	Climber	qí lín yè, qí lín wěi	WS-MD0028	33.33	St	Fresh	Dermal	For external use, pounded and applied as a poultice or decocted in water for washing.	Used to treat sprains and fractures.	Musculoskeletal Disorders
					66.67	St	Fresh	Dermal	For external use, pounded and applied as a poultice or decocted in water for washing.	Used to treat rheumatic pain.	Musculoskeletal Disorders
	Pothos chinensis (Raf.) Merr.	Herb	hú lu zuàn, pá shān wú gōng, shí gān zǐ	WS-MD0154	11.76	Wp	Dry, Fresh	Oral	Boiled in water and the filtrate is taken orally.	Used to treat rheumatic bone pain and traumatic injuries.	Musculoskeletal Disorders
					23.53	Wp	Dry, Fresh	Oral	Boiled in water and the filtrate is taken orally.	Used to treat cough.	Respiratory Disorders
					29.41	Wp	Dry, Fresh	Oral	Boiled in water and the filtrate is taken orally.	Used to treat infantile malnutrition.	Blood Disorders
					35.29	Wp	Dry, Fresh	Oral	Boiled in water and the filtrate is taken orally.	Used to treat postpartum edema.	Obstetrics, Gynaecology and Urinary Disorders
Araliaceae	*Dendropanax dentigerus* (Harms) Merr.	Shrub	yīn yáng fēng, fēng hé guì, shù shēn	WS-MD0059	6.67	Rt, St	Dry	Oral	Taken orally as a decoction or alcohol infusion.	Used to treat rheumatic pain.	Musculoskeletal Disorders
					20.00	Rt, St	Dry	Oral	Taken orally as a decoction or alcohol infusion.	Used to treat hemiplegia.	Musculoskeletal Disorders
					33.33	Rt, St	Dry	Oral	Taken orally as a decoction or alcohol infusion.	Used to treat migraine.	Central Nervous System Disorders

				40.00	Rt, St	Dry	Oral	Boiled in water and the filtrate is taken orally.	Used to treat irregular menstruation.	Obstetrics, Gynaecology and Urinary Disorders
Eleutherococcus nodiflorus (Dunn) S. Y. Hu	Shrub	wǔ jiā pí	WS-MD0025	33.33	Rt	Dry	Dermal	Soaked in alcohol and applied to the affected area.	Used to treat rheumatoid arthritis.	Musculoskeletal Disorders
				66.67	Rt	Dry	Dermal	Soaked in alcohol and applied to the affected area.	Used to treat sprains.	Musculoskeletal Disorders
Eleutherococcus trifoliatus (L.) S. Y. Hu	Shrub	jiǔ jì fēng, sān jiā pí	WS-MD0026	23.53	Rt	Dry, Fresh	Oral	Boiled in water and the filtrate is taken orally.	Used to treat stomach pain.	Gastrointestinal Disorders
				35.29	Rt	Dry, Fresh	Oral, Dermal	Taken orally as a decoction or alcohol infusion. For external use, pounded and applied to the affected area.	Used to treat rheumatoid arthritis.	Musculoskeletal Disorders
				41.18	Rt	Dry, Fresh	Oral, Dermal	Taken orally as a decoction or alcohol infusion. For external use, pounded and applied to the affected area.	Used to treat lower back and leg pain.	Musculoskeletal Disorders
Hedera sinensis (Tobler) Hand.-Mazz.	Climber	sān jiǎo fēng, cháng chūn téng	WS-MD0099	20.00	Wp	Dry, Fresh	Oral, Dermal	Boiled in water and the filtrate is taken orally. For external use, pounded and applied to the affected area.	Used to treat rheumatoid arthritis.	Musculoskeletal Disorders
				33.33	Wp	Dry, Fresh	Oral	Boiled in water and the filtrate is taken orally.	Used to treat hepatitis.	Gastrointestinal Disorders
				46.67	Wp	Dry, Fresh	Oral	Boiled in water and the filtrate is taken orally.	Used to treat inflammatory eye diseases.	Eye Disorders

	Heptapleurum heptaphyllum (L.) Y. F. Deng	Tree	yā jiǎo fēng, yā jiǎo mù	WS-MD0101	15.79	Bk	Dry, Fresh	Oral	Boiled in water and the filtrate is taken orally.	Used to treat colds, fever, and sore throat.	Infection/Immune Disorders
					26.32	Bk	Dry, Fresh	Oral, Dermal	Boiled in water and the filtrate is taken orally. For external use, pounded and applied to the affected area.	Used to treat rheumatic joint pain.	Musculoskeletal Disorders
					26.32	Bk	Dry, Fresh	Oral, Dermal	Boiled in water and the filtrate is taken orally. For external use, pounded and applied to the affected area.	Used to treat sprains and bruises.	Musculoskeletal Disorders
					31.58	Bk	Dry, Fresh	Oral, Dermal	Boiled in water and the filtrate is taken orally. For external use, pounded and applied to the affected area.	Used to treat fractures.	Musculoskeletal Disorders
	Hydrocotyle javanica Thunb.	Herb	hóng mă tí căo	WS-MD0104	31.25	Wp	Dry, Fresh	Oral, Dermal	Boiled in water and the filtrate is taken orally. For external use, pounded and applied to the affected area.	Used to treat injuries from falls and blows.	Musculoskeletal Disorders
					31.25	Wp	Dry, Fresh	Oral	Boiled in water and the filtrate is taken orally.	Used to treat colds.	Infection/Immune Disorders
					37.50	Wp	Dry, Fresh	Oral	Boiled in water and the filtrate is taken orally.	Used to treat cough with phlegm and blood.	Infection/Immune Disorders

	Tetrapanax papyrifer (Hook.) K.Koch	Tree	tōng tuō mù, yào yīng fēng, tōng cǎo	WS-MD0133	41.67	Ps	Dry	Oral	Boiled in water and the filtrate is taken orally.	Used to treat urinary retention.	Obstetrics, Gynaecology and Urinary Disorders
					58.33	Ps	Dry	Oral	Boiled in water and the filtrate is taken orally.	Used to promote lactation in postpartum women.	Obstetrics, Gynaecology and Urinary Disorders
Aristolochiaceae	*Asarum caudigerum* Hance	Herb	tǔ xì xīn, zhuī fēng cǎo	WS-MD0008	100.00	Wp	Dry, Fresh	Oral	Boiled in water and the filtrate is taken orally.	Used to treat colds and fever.	Infection/Immune Disorders
	Asarum geophilum Hemsl.	Herb	tǔ xì xīn, zhuī fēng cǎo	WS-MD0009	100.00	Wp	Dry, Fresh	Oral	Boiled in water and the filtrate is taken orally.	Used to treat colds and fever.	Infection/Immune Disorders
Asparagaceae	*Asparagus cochinchinensis* (Lour.) Merr.	Climber	tiān mén dōng	WS-MD0010	14.29	Tb	Dry, Fresh	Oral	Boiled in water and the filtrate is taken orally.	Used to treat colds and fever.	Infection/Immune Disorders
					14.29	Tb	Dry, Fresh	Oral	Boiled in water and the filtrate is taken orally.	Used to treat cough.	Respiratory Disorders
					14.29	Tb	Dry, Fresh	Oral	Boiled in water and the filtrate is taken orally.	Used to treat pneumonia.	Respiratory Disorders
					21.43	Tb	Dry, Fresh	Oral	Boiled in water and the filtrate is taken orally.	Used to treat postpartum anemia.	Blood Disorders
					35.71	Tb	Dry, Fresh	Oral	Boiled in water and the filtrate is taken orally.	Used to treat infertility.	Obstetrics, Gynaecology and Urinary Disorders
	Polygonatum cyrtonema Hua	Herb	tóng máo shuāng, huáng jīng, lǎo hǔ jiāng	WS-MD0151	47.37	Tb	Fresh, Processed	Oral	Taken orally as a decoction or stewed with lean pork and consumed.	Used to treat physical weakness.	Musculoskeletal Disorders
					15.79	Tb	Fresh, Processed	Oral	Taken orally as a decoction or stewed with lean pork and consumed.	Used to treat palpitations and shortness of breath.	Cardiological Disorders

					15.79	Tb	Fresh, Processed	Oral	Taken orally as a decoction or stewed with lean pork and consumed.	Used to treat dry cough due to lung dryness.	Respiratory Disorders
					21.05	Tb	Fresh, Processed	Oral	Taken orally as a decoction or stewed with lean pork and consumed.	Used to treat dry mouth due to prolonged illness.	Infection/Immune Disorders
	Polygonatum kingianum Collett & Hemsl.	Herb	huáng jīng, lǎo hǔ jiāng	WS-MD0152	47.62	Tb	Fresh, Processed	Oral	Taken orally as a decoction or stewed with lean pork and consumed.	Used to treat physical weakness.	Musculoskeletal Disorders
					19.05	Tb	Fresh, Processed	Oral	Taken orally as a decoction or stewed with lean pork and consumed.	Used to treat palpitations and shortness of breath.	Cardiological Disorders
					14.29	Tb	Fresh, Processed	Oral	Taken orally as a decoction or stewed with lean pork and consumed.	Used to treat dry cough due to lung dryness.	Respiratory Disorders
					19.05	Tb	Fresh, Processed	Oral	Taken orally as a decoction or stewed with lean pork and consumed.	Used to treat dry mouth due to prolonged illness.	Infection/Immune Disorders
Aspleniaceae	*Asplenium prolongatum* Hook.	Herb	shí shàng fēng, fèng huáng wěi	WS-MD0034	14.29	Wp	Fresh	Oral	Boiled in water and the filtrate is taken orally.	Used to treat pharyngitis.	Gastrointestinal Disorders
					14.29	Wp	Fresh	Oral	Boiled in water and the filtrate is taken orally.	Used to treat dysentery.	Infection/Immune Disorders
					19.05	Wp	Fresh	Oral	Boiled in water and the filtrate is taken orally.	Used to treat tuberculosis.	Infection/Immune Disorders

					23.81	Wp	Fresh	Dermal	For external use, an appropriate amount is pounded and applied to the affected area.	Used to treat burns and scalds.	Skin Disorders
					28.57	Wp	Fresh	Dermal	For external use, an appropriate amount is pounded and applied to the affected area.	Used to treat external bleeding.	Skin Disorders
Asteraceae	*Blumea balsamifera* (L.) DC.	Shrub	ài nà xiāng	WS-MD0037	17.65	Lv, St	Fresh	Oral	Boiled in water and the filtrate is taken orally.	Used to treat colds caused by wind and cold.	Respiratory Disorders
					17.65	Lv, St	Fresh	Dermal	For external use, pounded and applied to the affected area.	Used to treat rheumatic pain.	Musculoskeletal Disorders
					29.41	Lv, St	Fresh	Dermal	For external use, decoction for washing.	Used to treat eczema.	Skin Disorders
					35.29	Lv, St	Fresh	Dermal	For external use, decoction for washing.	Used to treat itchy skin.	Skin Disorders
	Blumea megacephala (Randeria) C.T.Chang & C.H.Yu	Herb	bái huā jiǔ lǐ míng, huá ài nà xiāng, dōng fēng cǎo	WS-MD0038	17.65	Wp	Fresh	Dermal	For external use, decoction for washing.	Used to treat rheumatic bone pain.	Musculoskeletal Disorders
					17.65	Wp	Fresh	Oral	Boiled in water and the filtrate is taken orally.	Used to treat irregular menstruation.	Obstetrics, Gynaecology and Urinary Disorders
					23.53	Wp	Fresh	Dermal	For external use, pounded and applied to the affected area.	Used to treat sprains and bruises.	Musculoskeletal Disorders
					41.18	Wp	Fresh	Dermal	For external use, pounded and applied to the affected area.	Used to treat swelling and pain from fractures.	Musculoskeletal Disorders

	Carpesium abrotanoides L.	Shrub	yě yān, yě yān yè	WS-MD0047	16.67	Wp	Fresh	Oral	Boiled in water and the filtrate is taken orally.	Used to treat sore throat.	Gastrointestinal Disorders
					16.67	Wp	Fresh	Oral	Boiled in water and the filtrate is taken orally.	Used to treat bronchitis.	Respiratory Disorders
					27.78	Wp	Fresh	Oral	Boiled in water and the filtrate is taken orally.	Used to treat hepatitis.	Gastrointestinal Disorders
					38.89	Wp	Fresh	Dermal	For external use, decoction for washing.	Used to treat sores and boils.	Skin Disorders
	Chrysanthemum indicum L.	Herb	yě jú	WS-MD0050	42.86	Ic	Dry	Oral	Boiled in water and the filtrate is taken orally.	Used to prevent influenza and colds.	Infection/Immune Disorders
					57.14	WP	Dry, Fresh	Oral	Boiled in water and the filtrate is taken orally.	Used to treat colds, fever, and pneumonia.	Infection/Immune Disorders
	Duhaldea cappa (Buch.-Ham. ex D.Don) Pruski & Anderb.	Shrub	bái miàn fēng, bái niú dǎn, guò shān xiāng	WS-MD0023	13.04	Wp	Fresh	Dermal	for external use, pounded and applied as a poultice or decocted in water for washing.	Used to treat rheumatic joint pain.	Musculoskeletal Disorders
					13.04	Wp	Dry, Fresh	Oral	Boiled in water and the filtrate is taken orally.	Used to treat diarrhea.	Infection/Immune Disorders
					17.39	Wp	Dry, Fresh	Oral	Boiled in water and the filtrate is taken orally.	Used to treat postpartum cold.	Infection/Immune Disorders
					26.09	Wp	Fresh	Dermal	For external use, decoction for washing.	Used to treat hemorrhoids.	Gastrointestinal Disorders
					30.43	Wp	Fresh	Dermal	For external use, decoction for washing.	Used to treat scabies.	Infection/Immune Disorders
	Elephantopus scaber L.	Herb	shā liè niàn, cǎo xié gēn, dì dǎn tóu	WS-MD0024	20.00	Wp	Dry, Fresh	Oral	Boiled in water and the filtrate is taken orally.	Used to treat stomach pain.	Gastrointestinal Disorders
					20.00	Wp	Dry, Fresh	Oral	Boiled in water and the filtrate is taken orally.	Used to treat tuberculosis.	Respiratory Disorders

					26.67	Wp	Dry, Fresh	Oral	Boiled in water and the filtrate is taken orally.	Used to treat cough.	Respiratory Disorders
					33.33	Wp	Dry, Fresh	Oral	Boiled in water and the filtrate is taken orally.	Used to treat toothache caused by wind and heat.	Gastrointestinal Disorders
	Gynura divaricata (L.) DC.	Herb	bái zǐ cài, bái bèi sān qī	WS-MD0098	11.11	Wp	Dry, Fresh	Oral	Boiled in water and the filtrate is taken orally.	Used to treat bronchitis.	Respiratory Disorders
					11.11	Wp	Dry, Fresh	Oral	Boiled in water and the filtrate is taken orally.	Used to treat infantile malnutrition.	Blood Disorders
					14.81	Wp	Dry, Fresh	Oral	Boiled in water and the filtrate is taken orally.	Used to treat red and swollen eyes.	Eye Disorders
					14.81	Wp	Fresh	Dermal	For external use, pounded and applied as a poultice or decocted in water for washing.	Used to treat mastitis.	Obstetrics, Gynaecology and Urinary Disorders
					22.22	Wp	Fresh	Dermal	For external use, pounded and applied to the affected area.	Used to treat sprains and bruises.	Musculoskeletal Disorders
					25.93	Wp	Fresh	Dermal	For external use, pounded and applied to the affected area.	Used to treat skin burns.	Skin Disorders
	Senecio scandens Buch.-Ham. ex D.Don	Herb	jiǔ lǐ míng, qiān lǐ guāng	WS-MD0172	27.27	Wp	Dry, Fresh	Oral	Boiled in water and the filtrate is taken orally.	Used to treat colds and fever.	Infection/Immune Disorders
					22.73	Wp	Dry, Fresh	Oral	Boiled in water and the filtrate is taken orally.	Used to treat cough.	Respiratory Disorders
					22.73	Wp	Fresh	Dermal	For external use, pounded and applied as a poultice or decocted in water for washing.	Used to treat eczema.	Skin Disorders

					13.64	Wp	Fresh	Dermal	For external use, pounded and applied as a poultice or decocted in water for washing.	Used to treat snake bites.	Poisoning/Toxicology
					13.64	Wp	Fresh	Dermal	For external use, pounded and applied as a poultice or decocted in water for washing.	Used to treat skin burns.	Skin Disorders
Balanophoraceae	*Balanophora fungosa* subsp. *indica* (Arn.) B.Hansen	Herb	shé gū, huán yáng cǎo	WS-MD0035	30.00	Wp	Dry, Fresh	Oral	Taken orally as a decoction or alcohol infusion	Used to treat sprains and bruises.	Musculoskeletal Disorders
					30.00	Wp	Dry, Fresh	Oral	Taken orally as a decoction or alcohol infusion	Used to treat hepatitis.	Gastrointestinal Disorders
					40.00	Wp	Dry, Fresh	Oral	Taken orally as a decoction or alcohol infusion	Used to treat hemoptysis.	Respiratory Disorders
Campanulaceae	*Codonopsis javanica* (Blume) Hook.f. & Thomson	Climber	tǔ dǎng shēn	WS-MD0054	71.43	Rt	Dry, Fresh	Oral	Boiled in water and the filtrate is taken orally.	Used to treat Qi deficiency and fatigue.	Musculoskeletal Disorders
					28.57	Rt	Dry, Fresh	Oral	Boiled in water and the filtrate is taken orally.	Used to treat neurasthenia.	Central Nervous System Disorders
Caprifoliaceae	*Lonicera hypoglauca* Miq.	Shrub	shān yín huā, jīn yín huā	WS-MD0119	40.00	Ic, Lv, St	Dry, Fresh	Oral	Boiled in water and the filtrate is taken orally.	Used to treat colds.	Infection/Immune Disorders
					40.00	Ic, Lv, St	Dry, Fresh	Oral	Boiled in water and the filtrate is taken orally.	Used to treat cough.	Respiratory Disorders
					20.00	Ic, Lv, St	Dry, Fresh	Dermal	For external use, decocted in water for washing.	Used to treat itchy skin.	Skin Disorders
	Lonicera macrantha (D.Don) Spreng.	Shrub	shān yín huā, jīn yín huā	WS-MD0120	40.00	Ic, Lv, St	Dry, Fresh	Oral	Boiled in water and the filtrate is taken orally.	Used to treat colds.	Infection/Immune Disorders
					40.00	Ic, Lv, St	Dry, Fresh	Oral	Boiled in water and the filtrate is taken orally.	Used to treat cough.	Respiratory Disorders

					20.00	Ic, Lv, St	Dry, Fresh	Dermal	For external use, decocted in water for washing.	Used to treat itchy skin.	Skin Disorders
Celastraceae	*Celastrus orbiculatus* Thunb.	Shrub	guò shān fēng, guò shān lóng, shān lóng, nán shé téng	WS-MD0048	15.79	St	Dry	Oral	Boiled in water and the filtrate is taken orally.	Used to treat muscle and bone pain.	Musculoskeletal Disorders
					26.32	St	Dry	Oral	Boiled in water and the filtrate is taken orally.	Used to treat numbness in the limbs.	Musculoskeletal Disorders
					26.32	St	Dry	Oral	Boiled in water and the filtrate is taken orally.	Used to treat infantile convulsions.	Musculoskeletal Disorders
					31.58	St	Dry	Oral	Boiled in water and the filtrate is taken orally.	Used to treat dysentery.	Infection/Immune Disorders
Chloranthaceae	*Sarcandra glabra* (Thunb.) Nakai	Herb	jiǔ jié fēng, jiǔ jié chá, zhǒng jié fēng, cǎo shān hú	WS-MD0165	13.04	Wp	Dry, Fresh	Oral	Boiled in water and the filtrate is taken orally.	Used to treat influenza.	Infection/Immune Disorders
					26.09	Wp	Dry, Fresh	Oral	Boiled in water and the filtrate is taken orally.	Used to treat pneumonia.	Respiratory Disorders
					21.74	Wp	Fresh	Dermal	For external use, pounded and applied to the affected area.	Used to treat sprains and bruises.	Musculoskeletal Disorders
					26.09	Wp	Dry, Fresh	Oral, Dermal	Boiled in water and the filtrate is taken orally. For external use, pounded and applied to the affected area.	Used to treat rheumatic joint pain.	Musculoskeletal Disorders
					13.04	Wp	Dry, Fresh	Oral	Boiled in water and the filtrate is taken orally.	Used to treat stomach cancer.	Cancer
Colchicaceae	*Disporum cantoniense* (Lour.) Merr.	Herb	wàn shòu zhú	WS-MD0020	31.58	Rz	Dry	Oral	Boiled in water and the filtrate is taken orally.	Used to treat cough associated with pulmonary tuberculosis.	Respiratory Disorders

					21.05	Rz	Dry	Oral	Boiled in water and the filtrate is taken orally.	Used to treat infantile malnutrition.	Blood Disorders
					21.05	Rz	Dry	Oral	Boiled in water and the filtrate is taken orally.	Used to treat lumbar muscle strain.	Musculoskeletal Disorders
					26.32	Rz	Dry	Oral	Boiled in water and the filtrate is taken orally.	Used to treat rheumatic bone pain.	Musculoskeletal Disorders
	Disporum trabeculatum Gagnep.	Herb	wàn shòu zhú	WS-MD0021	21.05	Rz	Dry	Oral	Boiled in water and the filtrate is taken orally.	Used to treat cough associated with pulmonary tuberculosis.	Respiratory Disorders
					21.05	Rz	Dry	Oral	Boiled in water and the filtrate is taken orally.	Used to treat infantile malnutrition.	Blood Disorders
					26.32	Rz	Dry	Oral	Boiled in water and the filtrate is taken orally.	Used to treat lumbar muscle strain.	Musculoskeletal Disorders
					31.58	Rz	Dry	Oral	Boiled in water and the filtrate is taken orally.	Used to treat rheumatic bone pain.	Musculoskeletal Disorders
Cornaceae	*Alangium chinense* (Lour.) Harms	Tree	bā jiǎo fēng	WS-MD0015	9.09	Rt	Dry	Oral	Taken orally as a decoction or alcohol infusion	Used to treat weakness.	Musculoskeletal Disorders
					18.18	Rt	Dry	Oral	Taken orally as a decoction or alcohol infusion	Used to treat cough.	Respiratory Disorders
					22.73	Rt	Dry	Oral	Taken orally as a decoction or alcohol infusion	Used to treat rheumatic bone pain.	Musculoskeletal Disorders
					22.73	Rt	Dry	Oral	Taken orally as a decoction or alcohol infusion	Used to treat numbness of the limbs.	Musculoskeletal Disorders
					27.27	Rt	Dry	Oral	Taken orally as a decoction or alcohol infusion	Used to treat injuries from falls and blows.	Musculoskeletal Disorders
Crassulaceae	*Kalanchoe pinnata* (Lam.) Pers.	Herb		WS-MD0112	23.08	Wp	Dry, Fresh	Oral	Boiled in water and the filtrate is taken orally.	Used to treat sore throat.	Gastrointestinal Disorders

			luò dì shēng gēn, yè shēng gēn		30.77	Wp	Fresh	Dermal	For external use, pounded and applied as a poultice.	Used to treat bruises and skin burns.	Skin Disorders
					46.15	Wp	Fresh	Dermal	For external use, pounded and applied as a poultice.	Used to treat scalds.	Skin Disorders
Cucurbitaceae	*Gynostemma pentaphyllum* (Thunb.) Makino	Climber	qī yè dǎn, jiǎo gǔ lán	WS-MD0097	25.00	Wp	Dry, Fresh	Oral	Boiled in water and the filtrate is taken orally.	Used to treat bronchitis.	Respiratory Disorders
					20.83	Wp	Dry, Fresh	Ora	Boiled in water and the filtrate is taken orally.	Used to treat pharyngitis.	Gastrointestinal Disorders
					12.50	Wp	Dry, Fresh	Oral	Boiled in water and the filtrate is taken orally.	Used to treat gastroenteritis.	Gastrointestinal Disorders
					16.67	Wp	Dry, Fresh	Oral	Boiled in water and the filtrate is taken orally.	Used to treat tumors.	Cancer
					25.00	Wp	Fresh	Dermal	For external use, pounded and applied as a poultice.	Used to treat snake bites.	Poisoning/Toxicology
Dioscoreaceae	*Dioscorea bulbifera* L.	Climber	huáng dú, huáng yào zǐ	WS-MD0019	25.00	Bb, Tb	Dry	Oral	Slightly toxic; decoction taken in appropriate amounts.	Used to treat whooping cough.	Respiratory Disorders
					37.50	Bb, Tb	Dry	Oral	Slightly toxic; decoction taken in appropriate amounts.	Used to treat acute and chronic bronchitis.	Respiratory Disorders
					37.50	Bb, Tb	Fresh	Dermal	For external use, pounded and applied as a poultice.	Used to treat snake bites.	Poisoning/Toxicology
Equisetaceae	*Equisetum ramosissimum* var. *huegelii* (Milde) Christenh. & Husby	Herb	bā tào mī, mù zéi, bǐ tǒng cǎo, mù zéi cǎo	WS-MD0029	100.00	Wp	Dry, Fresh	Oral	Boiled in water and the filtrate is taken orally.	Used to treat red, swollen, and painful eyes.	Eye Disorders

Ericaceae	*Gaultheria crenulata* Kurz	Shrub	xià shān hǔ, diān bái zhū, mǎn shān xiāng	WS-MD0093	31.58	Wp	Dry	Oral, Dermal	Boiled in water and the filtrate is taken orally. For external use, pounded and applied as a poultice or decocted in water for washing.	Used to treat rheumatoid arthritis.	Musculoskeletal Disorders
					26.32	Wp	Dry	Oral, Dermal	Boiled in water and the filtrate is taken orally. For external use, pounded and applied as a poultice or decocted in water for washing.	Used to treat sprains and bruises.	Musculoskeletal Disorders
					15.79	Wp	Dry	Oral	Boiled in water and the filtrate is taken orally.	Used to treat chronic bronchitis.	Respiratory Disorders
					26.32	Wp	Dry	Oral	Boiled in water and the filtrate is taken orally.	Used to treat colds caused by wind and cold.	Infection/Immune Disorders
Euphorbiaceae	*Alchornea trewioides* (Benth.) Müll.Arg.	Shrub	hóng mào dǐng, hóng bèi yè, hóng bèi niáng	WS-MD0016	15.79	Rt	Dry	Oral	Boiled in water and the filtrate is taken orally.	Used to treat enteritis and diarrhea.	Gastrointestinal Disorders
					21.05	Rt	Dry	Oral	Boiled in water and the filtrate is taken orally.	Used to treat jaundice hepatitis.	Gastrointestinal Disorders
					31.58	Rt	Dry	Oral	Boiled in water and the filtrate is taken orally.	Used to treat urinary tract infections.	Obstetrics, Gynaecology and Urinary Disorders
					31.58	Rt	Dry	Oral	Boiled in water and the filtrate is taken orally.	Used to treat kidney stones.	Obstetrics, Gynaecology and Urinary Disorders
	Euphorbia hirta L.	Herb	dà fēi yáng cǎo, máo fēi	WS-MD0031	20.00	Wp	Dry, Fresh	Oral	Boiled in water and the filtrate is taken orally.	Used to treat enteritis and dysentery.	Gastrointestinal Disorders

			yáng, dà rǔ zhī cǎo, nǎi zi cǎo		25.00	Wp	Dry, Fresh	Oral	Boiled in water and the filtrate is taken orally.	Used to treat postpartum hypogalactia.	Obstetrics, Gynaecology and Urinary Disorders
					25.00	Wp	Fresh	Dermal	For external use, pounded and applied as a poultice or decocted in water for washing.	Used to treat carbuncles and boils.	Infection/Immune Disorders
					30.00	Wp	Fresh	Dermal	For external use, pounded and applied as a poultice or decocted in water for washing.	Used to treat eczema and dermatitis.	Skin Disorders
	Euphorbia thymifolia L.	Herb	xiǎo fēi yáng, xì yè xiǎo jǐn cǎo	WS-MD0032	19.05	Wp	Dry, Fresh	Oral	Boiled in water and the filtrate is taken orally.	Used to treat infantile malnutrition.	Blood Disorders
					23.81	Wp	Dry, Fresh	Oral	Boiled in water and the filtrate is taken orally.	Used to treat dysentery.	Infection/Immune Disorders
					28.57	Wp	Fresh	Dermal	For external use, pounded and applied as a poultice or decocted in water for washing.	Used to treat eczema.	Skin Disorders
					28.57	Wp	Fresh	Dermal	For external use, pounded and applied as a poultice or decocted in water for washing.	Used to treat mastitis.	Obstetrics, Gynaecology and Urinary Disorders
	Mallotus apelta (Lour.) Müll.Arg.	Tree	bái diào lì, bái bèi niáng, bái yè yě tóng	WS-MD0067	11.54	Rt	Dry	Oral	Boiled in water and the filtrate is taken orally.	Used to treat hepatitis.	Gastrointestinal Disorders
					19.23	Rt	Dry	Oral	Boiled in water and the filtrate is taken orally.	Used to treat kidney deficiency.	Obstetrics, Gynaecology and Urinary Disorders
					19.23	Rt	Dry	Oral	Boiled in water and the filtrate is taken orally.	Used to treat lower back pain.	Musculoskeletal Disorders

					23.08	Rt	Dry	Oral	Boiled in water and the filtrate is taken orally.	Used to treat uterine prolapse.	Obstetrics, Gynaecology and Urinary Disorders
					26.92	Rt	Dry	Oral	Boiled in water and the filtrate is taken orally.	Used to treat rectal prolapse.	Gastrointestinal Disorders
Fabaceae	*Bauhinia brachycarpa* Wall. ex Benth.	Climber	hú dié fēng, tí téng, mù yè guān mén	WS-MD0036	12.50	Rt	Dry	Oral	Taken orally as a decoction or alcohol infusion	Used to treat hemiplegia.	Musculoskeletal Disorders
					16.67	Rt	Dry	Oral	Boiled in water and the filtrate is taken orally.	Used to treat whooping cough.	Respiratory Disorders
					20.83	Rt	Dry	Oral	Boiled in water and the filtrate is taken orally.	Used to treat dysentery.	Infection/Immune Disorders
					25.00	Rt	Dry	Oral	Boiled in water and the filtrate is taken orally.	Used to treat diarrhea.	Infection/Immune Disorders
					25.00	Rt	Dry	Oral	Taken orally as a decoction or alcohol infusion	Used to treat rheumatic pain.	Musculoskeletal Disorders
	Callerya cinerea (Benth.) Schot	Climber	jī xuè téng	WS-MD0041	21.74	St	Dry	Oral	Taken orally as a decoction or alcohol infusion	Used to treat anemia.	Blood Disorders
					21.74	St	Dry	Oral	Taken orally as a decoction or alcohol infusion	Used to treat dizziness.	Central Nervous System Disorders
					26.09	St	Dry	Oral	Taken orally as a decoction or alcohol infusion	Used to treat irregular menstruation.	Obstetrics, Gynaecology and Urinary Disorders
					30.43	St	Dry	Oral	Taken orally as a decoction or alcohol infusion	Used to treat rheumatic pain.	Musculoskeletal Disorders
	Crotalaria pallida Aiton	Herb	zhū shǐ dòu	WS-MD0055	45.45	Lv	Dry	Oral	Boiled in water and the filtrate is taken orally.	Used to treat high blood pressure.	Blood Disorders
					54.55	Lv	Dry	Oral	Boiled in water and the filtrate is taken orally.	Used to treat diabetes.	Blood Disorders

Entada phaseoloides (L.) Merr.	Climber	niǔ gǔ fēng, guò jiāng lóng	WS-MD0027	100.00	St	Dry	Oral	Taken orally as a decoction or alcohol infusion	Used to treat rheumatic bone pain.	Musculoskeletal Disorders
Flemingia macrophylla (Willd.) Kuntze ex Merr.	Shrub	dà yè qiān jīn bá, qiān jīn lì	WS-MD0090	46.15	Rt	Dry	Oral, Dermal	Taken as a decoction, or soaked in alcohol for oral use, and also used topically after soaking in alcohol.	Used to treat rheumatoid arthritis.	Musculoskeletal Disorders
				23.08	Rt	Dry	Oral	Taken as a decoction, or soaked in alcohol for oral use.	Used to treat chronic bronchitis.	Respiratory Disorders
				30.77	Rt	Dry	Oral	Taken as a decoction, or soaked in alcohol for oral use.	Used to treat cough due to overwork.	Respiratory Disorders
Flemingia prostrata Roxb. Junior ex Roxb.	Herb	qiān jīn bá, dà lì huáng	WS-MD0091	100.00	Rt	Dry	Oral, Dermal	Taken as a decoction, or soaked in alcohol for oral use, and also used topically after soaking in alcohol.	Used to treat rheumatic pain and rheumatoid arthritis.	Musculoskeletal Disorders
Guilandina minax (Hance) G.P.Lewis	Shrub	nán shé lè, nán shé fēng, shí lián téng, shí lián zǐ	WS-MD0096	45.45	Sd	Dry	Oral	Boiled in water and the filtrate is taken orally.	Used to treat acute gastroenteritis.	Gastrointestinal Disorders
				27.27	Sd	Dry	Oral	Boiled in water and the filtrate is taken orally.	Used to treat dysentery.	Infection/Immune Disorders
				27.27	Sd	Dry	Oral	Boiled in water and the filtrate is taken orally.	Used to treat cystitis.	Obstetrics, Gynaecology and Urinary Disorders
Kummerowia striata (Thunb.) Schindl.	Herb	jī yǎn cǎo, cāng ying cǎo, rén zì cǎo	WS-MD0113	25.00	Wp	Dry, Fresh	Oral	Boiled in water and the filtrate is taken orally.	Used to treat colds and cough.	Infection/Immune Disorders
				33.33	Wp	Dry, Fresh	Oral	Boiled in water and the filtrate is taken orally.	Used to treat fever.	Infection/Immune Disorders

					41.67	Wp	Fresh	Dermal	For external use, pounded and applied as a poultice or decocted in water for washing.	Used to treat injuries from falls and blows.	Musculoskeletal Disorders
	Mucuna birdwoodiana Tutcher	Climber	bái huā yóu má téng, yóu má téng	WS-MD0070	31.58	St	Dry	Oral	Taken orally as a decoction or alcohol infusion.	Used to treat rheumatic bone pain.	Musculoskeletal Disorders
					31.58	St	Dry	Oral	Taken orally as a decoction or alcohol infusion.	Used to treat lower back and knee pain.	Musculoskeletal Disorders
					36.84	St	Dry	Oral	Taken orally as a decoction or alcohol infusion.	Used to treat irregular menstruation.	Obstetrics, Gynaecology and Urinary Disorders
	Mucuna sempervirens Hemsl.	Climber	yóu má téng	WS-MD0071	27.78	St	Dry	Oral	Taken orally as a decoction or alcohol infusion.	Used to treat rheumatic bone pain.	Musculoskeletal Disorders
					33.33	St	Dry	Oral	Taken orally as a decoction or alcohol infusion.	Used to treat lower back and knee pain.	Musculoskeletal Disorders
					38.89	St	Dry	Oral	Taken orally as a decoction or alcohol infusion.	Used to treat irregular menstruation.	Obstetrics, Gynaecology and Urinary Disorders
	Phanera championii Benth.	Climber	jiǔ lóng zuān, jiǔ lóng téng, yàn zǐ wěi	WS-MD0080	45.45	St	Dry	Oral	Taken orally as a decoction or alcohol infusion.	Used to treat rheumatoid arthritis.	Musculoskeletal Disorders
					54.55	St	Dry	Dermal	Used topically after soaking in alcohol.	Used to treat sprains.	Musculoskeletal Disorders
	Phyllodium pulchellum (L.) Desv.	Climber	jīn qián fēng, lóng lín cǎo, qián pái cǎo	WS-MD0086	18.75	Wp	Dry, Fresh	Oral	Taken orally as a decoction or alcohol infusion.	Used to treat colds.	Infection/Immune Disorders
					18.75	Wp	Dry, Fresh	Oral	Taken orally as a decoction or alcohol infusion.	Used to treat rheumatic pain.	Musculoskeletal Disorders

					25.00	Wp	Dry, Fresh	Oral	Taken orally as a decoction or alcohol infusion.	Used to treat toothache and swelling.	Gastrointestinal Disorders
					37.50	Wp	Dry, Fresh	Oral	Taken orally as a decoction or alcohol infusion.	Used to treat pain from falls and injuries.	Musculoskeletal Disorders
	Pueraria montana (Lour.) Merr.	Climber	wǔ céng fēng, gě gēn	WS-MD0158	42.86	Rt	Dry	Oral	Boiled in water and the filtrate is taken orally.	Used to treat colds with fever and fever with thirst.	Infection/Immune Disorders
					57.14	Rt	Dry	Oral	Boiled in water and the filtrate is taken orally.	Used to treat difficulty urinating.	Obstetrics, Gynaecology and Urinary Disorders
	Senna tora (L.) Roxb.	Herb	yè tū mī, cǎo jué míng, jué míng	WS-MD0173	29.41	Sd	Dry	Oral	Boiled in water and the filtrate is taken orally.	Used to treat colds.	Infection/Immune Disorders
					17.65	Sd	Dry	Oral	Boiled in water and the filtrate is taken orally.	Used to treat hepatitis.	Gastrointestinal Disorders
					17.65	Sd	Dry	Oral	Boiled in water and the filtrate is taken orally.	Used to treat high blood pressure.	Blood Disorders
					17.65	Sd	Dry	Oral	Boiled in water and the filtrate is taken orally.	Used to treat constipation.	Gastrointestinal Disorders
					17.65	Sd	Dry	Oral	Boiled in water and the filtrate is taken orally.	Used to treat infantile malnutrition.	Blood Disorders
	Spatholobus suberectus Dunn	Climber	jiǔ céng fēng, jī xuè téng, sān yè jī xuè téng	WS-MD0125	25.00	St	Dry	Oral	Boiled in water and the filtrate is taken orally.	Used to treat anemia.	Blood Disorders
					15.00	St	Dry	Oral	Boiled in water and the filtrate is taken orally.	Used to treat dizziness.	Central Nervous System Disorders
					30.00	St	Dry	Oral	Boiled in water and the filtrate is taken orally.	Used to treat irregular menstruation.	Obstetrics, Gynaecology and Urinary Disorders

					30.00	St	Dry	Oral	Boiled in water and the filtrate is taken orally.	Used to treat rheumatic pain.	Musculoskeletal Disorders
	Tadehagi triquetrum (L.) H.Ohashi	Herb	gǔ lù chá, hú lu chá	WS-MD0128	16.00	Wp	Dry, Fresh	Oral	Boiled in water and the filtrate is taken orally.	Used to treat colds with fever.	Infection/Immune Disorders
					16.00	Wp	Dry, Fresh	Oral	Boiled in water and the filtrate is taken orally.	Used to treat heatstroke.	Central Nervous System Disorders
					20.00	Wp	Dry, Fresh	Oral	Boiled in water and the filtrate is taken orally.	Used to treat sore throat.	Gastrointestinal Disorders
					24.00	Wp	Dry, Fresh	Oral	Boiled in water and the filtrate is taken orally.	Used to treat dysentery.	Infection/Immune Disorders
					24.00	Wp	Fresh	Dermal	For external use, decocted in water for washing.	Used to treat skin ulcers.	Skin Disorders
Gentianaceae	*Tripterospermum chinense* (Migo) Harry Sm.	Climber	shuāng hú dié, máo jīn měi, fèi xíng cǎo	WS-MD0137	46.67	Wp	Dry, Fresh	Oral	Boiled in water and the filtrate is taken orally.	Used to treat bronchitis.	Respiratory Disorders
					53.33	Wp	Dry, Fresh	Oral	Boiled in water and the filtrate is taken orally.	Used to treat infantile febrile convulsions.	Infection/Immune Disorders
Gnetaceae	*Gnetum montanum* Markgr.	Climber	má gǔ zuàn	WS-MD0095	25.00	St	Dry	Oral	Boiled in water and the filtrate is taken orally.	Used to treat sprains.	Musculoskeletal Disorders
					33.33	St	Dry	Oral	Boiled in water and the filtrate is taken orally.	Used to treat lumbar muscle strain.	Musculoskeletal Disorders
					41.67	St	Dry	Oral	Boiled in water and the filtrate is taken orally.	Used to treat rheumatic bone pain.	Musculoskeletal Disorders
Hypericaceae	*Hypericum japonicum* Thunb.	Herb	tián jī huáng	WS-MD0105	13.04	Wp	Dry	Oral	Boiled in water and the filtrate is taken orally.	Used to treat hepatitis.	Gastrointestinal Disorders
					17.39	Wp	Dry	Oral	Boiled in water and the filtrate is taken orally.	Used to treat pharyngitis.	Gastrointestinal Disorders
					21.74	Wp	Dry	Oral	Boiled in water and the filtrate is taken orally.	Used to treat diarrhea.	Infection/Immune Disorders

					21.74	Wp	Dry	Oral	Boiled in water and the filtrate is taken orally.	Used to treat infantile malnutrition.	Blood Disorders
					26.09	Wp	Dry	Oral	Boiled in water and the filtrate is taken orally.	Used to treat tonsillitis.	Respiratory Disorders
	Hypericum sampsonii Hance	Herb	yuán bǎo mī, fān chuán cǎo, yè bào zhī	WS-MD0106	18.18	Wp	Dry, Fresh	Oral	Boiled in water and the filtrate is taken orally.	Used to treat irregular menstruation.	Obstetrics, Gynaecology and Urinary Disorders
					22.73	Wp	Dry, Fresh	Oral, Dermal	Taken orally as a decoction; for external use, pounded and applied as a poultice.	Used to treat injuries from falls and blows.	Musculoskeletal Disorders
					27.27	Wp	Dry, Fresh	Oral, Dermal	Taken orally as a decoction; for external use, pounded and applied as a poultice.	Used to treat boils and carbuncles.	Infection/Immune Disorders
					31.82	Wp	Dry, Fresh	Oral, Dermal	Taken orally as a decoction; for external use, pounded and applied as a poultice.	Used to treat snake bites.	Poisoning/Toxicology
Hypoxidaceae	*Curculigo capitulata* (Lour.) Kuntze	Herb	tóng móu zhōng, dà yè xiān máo, dà dì zōng gēn	WS-MD0056	22.22	Rz	Dry	Oral	Boiled in water and the filtrate is taken orally.	Used to treat cough due to kidney deficiency.	Obstetrics, Gynaecology and Urinary Disorders
					33.33	Rz	Dry	Oral	Boiled in water and the filtrate is taken orally.	Used to treat chronic bronchitis.	Respiratory Disorders
					44.44	Rz	Fresh	Dermal	For external use, pounded and applied as a poultice or decocted in water for washing.	Used to treat rheumatoid arthritis.	Musculoskeletal Disorders

	Curculigo orchioides Gaertn.	Herb	móu zhōng, dú jiǎo xiān máo, xiān máo	WS-MD0057	19.05	Rz	Dry	Oral	Taken orally as a decoction or alcohol infusion.	Used to treat cold pain in the chest and abdomen.	Cardiological Disorders
					23.81	Rz	Dry	Oral	Taken orally as a decoction or alcohol infusion.	Used to treat gastroptosis.	Gastrointestinal Disorders
					28.57	Rz	Dry	Oral	Taken orally as a decoction or alcohol infusion.	Used to treat impotence.	Reproductive Disorders
					28.57	Rz	Dry, Fresh	Oral, Dermal	Taken orally as a decoction or alcohol infusion. for external use, pounded and applied as a poultice or decocted in water for washing.	Used to treat rheumatic pain in the muscles and bones.	Musculoskeletal Disorders
Icacinaceae	*Mappianthus iodoides* Hand.-Mazz.	Climber	tóng zuàn, dìng xīn téng	WS-MD0068	15.38	Rt, St	Dry	Oral	Taken orally as a decoction or alcohol infusion.	Used to treat rheumatic pain and rheumatoid arthritis.	Musculoskeletal Disorders
					15.38	Rt, St	Dry	Oral	Boiled in water and the filtrate is taken orally.	Used to treat menstrual disorders.	Obstetrics, Gynaecology and Urinary Disorders
					38.46	Rt, St	Fresh	Dermal	For external use, pounded and applied to the affected area.	Used to treat traumatic bleeding.	Skin Disorders
					30.77	Rt, St	Dry	Oral	Boiled in water and the filtrate is taken orally.	Used to treat colds, fever, and cough.	Infection/Immune Disorders
Lamiaceae	*Anisomeles indica* (L.) Kuntze	Herb	fáng fēng mī, guǎng fáng fēng, tǔ huò xiāng	WS-MD0002	42.86	Lv, St	Fresh	Dermal	For external use, pounded and applied as a poultice or decocted in water for washing.	Used to treat snake and insect bites.	Poisoning/Toxicology

					57.14	Lv, St	Dry, Fresh	Oral	Boiled in water and the filtrate is taken orally.	Used to treat colds.	Infection/Immune Disorders
	Callicarpa bodinieri H.Lév.	Shrub	zhēn zhū fēng, dà yè bān jiū mǐ, zǐ zhū	WS-MD0042	7.14	Lv	Dry, Fresh	Dermal	For external use, pounded and applied as a poultice or decocted in water for washing.	Used to treat rheumatic pain.	Musculoskeletal Disorders
					10.71	Lv	Dry, Fresh	Oral	Boiled in water and the filtrate is taken orally.	Used to treat metrorrhagia.	Obstetrics, Gynaecology and Urinary Disorders
					14.29	Lv	Dry, Fresh	Oral	Boiled in water and the filtrate is taken orally.	Used to treat irregular menstruation.	Obstetrics, Gynaecology and Urinary Disorders
					14.29	Lv	Fresh	Dermal	For external use, pounded and applied to the affected area.	Used to treat external bleeding.	Skin Disorders
					17.86	Lv	Fresh	Dermal	For external use, Applied as a decocted in water for washing.	Used to treat itchy skin.	Skin Disorders
	Callicarpa macrophylla Vahl	Shrub	chuān gǔ fēng, dà fēng yè, bái gǔ fēng	WS-MD0044	14.81	Wp	Dry, Fresh	Oral	Boiled in water and the filtrate is taken orally.	Used to treat bleeding after tooth extraction.	Gastrointestinal Disorders
					18.52	Wp	Fresh	Dermal	For external use, pounded and applied as a poultice or decocted in water for washing.	Used to treat swelling and pain from falls and injuries.	Musculoskeletal Disorders
					18.52	Wp	Dry, Fresh	Oral, Dermal	Taken orally as a decoction; for external use, pounded and applied as a poultice or decocted in water for washing.	Used to treat rheumatic bone pain.	Musculoskeletal Disorders

					22.22	Wp	Dry, Fresh	Oral	Boiled in water and the filtrate is taken orally.	Used to treat tuberculosis cough.	Respiratory Disorders
					25.93	Wp	Dry, Fresh	Oral, Dermal	Taken orally as a decoction; for external use, pounded and applied as a poultice or decocted in water for washing.	Used to treat rheumatism.	Musculoskeletal Disorders
	Callicarpa rubella Lindl.	Shrub	yě yě pèng, hóng zǐ zhū, jié jié fēng	WS-MD0045	46.67	Rt	Dry, Fresh	Oral, Dermal	Taken orally as a decoction; for external use, pounded and applied as a poultice.	Used to treat rheumatism.	Musculoskeletal Disorders
					53.33	Lv	Fresh	Dermal	For external use, pounded and applied as a poultice.	Used to treat external bleeding.	Skin Disorders
	Clerodendrum japonicum (Thunb.) Sweet	Shrub	hóng dǐng fēng, hóng lóng chuán huā	WS-MD0052	16.00	Wp	Dry, Fresh	Oral	Boiled in water and the filtrate is taken orally.	Used to treat irregular menstruation.	Obstetrics, Gynaecology and Urinary Disorders
					16.00	Wp	Dry, Fresh	Oral	Boiled in water and the filtrate is taken orally.	Used to treat uterine prolapse.	Obstetrics, Gynaecology and Urinary Disorders
					20.00	Wp	Dry, Fresh	Oral, Dermal	Taken orally as a decoction; for external use, pounded and applied as a poultice or decocted in water for washing.	Used to treat rheumatic bone pain.	Musculoskeletal Disorders
					20.00	Wp	Dry, Fresh	Oral	Boiled in water and the filtrate is taken orally.	Used to treat dysentery.	Infection/Immune Disorders
					28.00	Wp	Dry, Fresh	Oral	Boiled in water and the filtrate is taken orally.	Used to treat colds.	Infection/Immune Disorders

	Clerodendrum lindleyi Decne. ex Planch.	Shrub	guǐ diǎn huǒ, chòu mǔ dān, chòu mò lì	WS-MD0053	14.29	Wp	Dry	Oral	Boiled in water and the filtrate is taken orally.	Used to treat menstrual disorders.	Obstetrics, Gynaecology and Urinary Disorders
					14.29	Wp	Dry, Fresh	Oral, Dermal	Taken orally as a decoction; for external use, pounded and applied as a poultice or decocted in water for washing.	Used to treat rheumatic bone pain.	Musculoskeletal Disorders
					28.57	Wp	Dry, Fresh	Oral, Dermal	Taken orally as a decoction; for external use, pounded and applied as a poultice or decocted in water for washing.	Used to treat fractures.	Musculoskeletal Disorders
					42.86	Wp	Dry	Oral	Boiled in water and the filtrate is taken orally.	Used to treat otitis media in women.	Infection/Immune Disorders
	Glechoma longituba (Nakai) Kuprian.	Herb	zuàn dì fēng, tòu gǔ xiāo, mǎ tí cǎo, huó xuè dān	WS-MD0094	37.50	Lv, St	Dry, Fresh	Oral	Boiled in water and the filtrate is taken orally.	Used to treat urinary tract stones and liver and gallbladder stones.	Obstetrics, Gynaecology and Urinary Disorders
					62.50	Lv, St	Fresh	Dermal	For external use, pounded and applied as a poultice or decocted in water for washing.	Used to treat sprains and bruises.	Musculoskeletal Disorders
	Leonurus japonicus Houtt.	Herb	yì mǔ ài, yì mǔ cǎo	WS-MD0115	33.33	Wp	Dry, Fresh	Oral	Boiled in water and the filtrate is taken orally.	Used to treat irregular menstruation.	Obstetrics, Gynaecology and Urinary Disorders
					19.05	Wp	Dry, Fresh	Oral	Boiled in water and the filtrate is taken orally.	Used to treat postpartum abdominal pain.	Obstetrics, Gynaecology and Urinary Disorders

					28.57	Wp	Dry, Fresh	Oral, Dermal	Taken orally as a decoction; for external use, pounded and applied as a poultice or decocted in water for washing.	Used to treat sprains and bruises.	Musculoskeletal Disorders
					19.05	Wp	Fresh	Dermal	For external use, pounded and applied to the affected area.	Used to treat snake bites.	Poisoning/Toxicology
	Perilla frutescens (L.) Britton	Herb	zǐ sū	WS-MD0077	40.00	Lv, St	Dry	Oral	Boiled in water and the filtrate is taken orally.	Used to treat colds.	Infection/Immune Disorders
					60.00	Lv, St	Dry	Oral	Boiled in water and the filtrate is taken orally.	Used to treat cough.	Respiratory Disorders
	Prunella vulgaris L.	Herb	zǐ huā cǎo, xià kū cǎo	WS-MD0155	6.67	Wp	Dry	Oral	Boiled in water and the filtrate is taken orally.	Used to treat hypertension.	Blood Disorders
					6.67	Wp	Dry	Oral	Boiled in water and the filtrate is taken orally.	Used to treat headache.	Central Nervous System Disorders
					10.00	Wp	Dry	Oral	Boiled in water and the filtrate is taken orally.	Used to treat dizziness.	Central Nervous System Disorders
					13.33	Wp	Dry	Oral	Boiled in water and the filtrate is taken orally.	Used to treat tuberculosis.	Respiratory Disorders
					20.00	Wp	Dry	Oral	Boiled in water and the filtrate is taken orally.	Used to treat urethritis.	Obstetrics, Gynaecology and Urinary Disorders
					20.00	Wp	Dry	Oral	Boiled in water and the filtrate is taken orally.	Used to treat cystitis.	Obstetrics, Gynaecology and Urinary Disorders
					23.33	Wp	Dry	Oral	Boiled in water and the filtrate is taken orally.	Used to treat nephritis.	Obstetrics, Gynaecology and Urinary Disorders

Lardiza-balaceae	*Akebia trifoliata* (Thunb.) Koidz.	Climber	lán jiǔ niú, sān yè mù tōng, bā yuè guā	WS-MD0014	35.71	Rt	Dry	Oral	Taken orally as a decoction or alcohol infusion	Used to treat rheumatoid arthritis.	Musculoskeletal Disorders
					35.71	Rt	Dry	Oral	Taken orally as a decoction or alcohol infusion	Used to treat lower back pain.	Musculoskeletal Disorders
					28.57	Rt	Dry	Oral	Taken orally as a decoction or alcohol infusion	Used to treat sprains and bruises.	Musculoskeletal Disorders
	Sargentodoxa cuneata (Oliv.) Rehder & E.H.Wilson	Climber	bīng láng zuān, dà xuè téng, dà huó xuè	WS-MD0166	28.00	St	Dry	Oral	Boiled in water and the filtrate is taken orally.	Used to treat rheumatoid arthritis.	Musculoskeletal Disorders
					24.00	St	Dry	Oral	Boiled in water and the filtrate is taken orally.	Used to treat numbness of the limbs.	Musculoskeletal Disorders
					12.00	St	Dry	Oral	Boiled in water and the filtrate is taken orally.	Used to treat sprains and bruises.	Musculoskeletal Disorders
					16.00	St	Dry	Oral	Boiled in water and the filtrate is taken orally.	Used to treat irregular menstruation.	Obstetrics, Gynaecology and Urinary Disorders
					12.00	St	Dry	Oral	Boiled in water and the filtrate is taken orally.	Used to treat infantile malnutrition.	Blood Disorders
					8.00	St	Dry	Oral	Boiled in water and the filtrate is taken orally.	Used to treat indigestion.	Gastrointestinal Disorders
Lauraceae	*Camphora migao* (H. W. Li) Y. Yang, Bing Liu & Zhi Yang	Tree	mǐ gǎo, dà guǒ zhāng, má gào	WS-MD0046	100.00	Ft	Dry	Oral	Eat raw.	Used to treat stomach ache.	Gastrointestinal Disorders
Liliaceae	*Lilium brownii* Lemoinier	Herb	yě bǎi hé	WS-MD0117	38.46	Bl	Dry, Fresh	Oral	Boiled in water and the filtrate is taken orally.	Used to treat lung heat cough and bronchitis.	Respiratory Disorders

					23.08	Bl	Dry, Fresh	Oral	Taken orally as a decoction, steamed, or cooked into porridge.	Used to treat neurasthenia.	Central Nervous System Disorders
					38.46	Bl	Dry, Fresh	Oral	Taken orally as a decoction, steamed, or cooked into porridge.	Used to treat insomnia.	Central Nervous System Disorders
Loranthaceae	*Helixanthera parasitica* Lour.	Shrub	jì shēng	WS-MD0100	42.86	Wp	Dry	Oral	Boiled in water and the filtrate is taken orally.	Used to treat rheumatic pain.	Musculoskeletal Disorders
					35.71	Wp	Dry	Oral	Boiled in water and the filtrate is taken orally.	Used to treat lower back and knee pain.	Musculoskeletal Disorders
					21.43	Wp	Dry	Oral	Boiled in water and the filtrate is taken orally.	Used to treat sprains and bruises.	Musculoskeletal Disorders
	Macrosolen cochinchinensis (Lour.) Tiegh.	Shrub	jì shēng	WS-MD0066	28.57	Wp	Dry	Oral	Boiled in water and the filtrate is taken orally.	Used to treat rheumatic pain.	Musculoskeletal Disorders
					35.71	Wp	Dry	Oral	Boiled in water and the filtrate is taken orally.	Used to treat lower back and knee pain.	Musculoskeletal Disorders
					35.71	Wp	Dry	Oral	Boiled in water and the filtrate is taken orally.	Used to treat sprains and bruises.	Musculoskeletal Disorders
	Scurrula parasitica L.	Shrub	jì shēng	WS-MD0169	46.15	Wp	Dry	Oral	Boiled in water and the filtrate is taken orally.	Used to treat rheumatic pain.	Musculoskeletal Disorders
					30.77	Wp	Dry	Oral	Boiled in water and the filtrate is taken orally.	Used to treat lower back and knee pain.	Musculoskeletal Disorders
					23.08	Wp	Dry	Oral	Boiled in water and the filtrate is taken orally.	Used to treat sprains and bruises.	Musculoskeletal Disorders

	Taxillus chinensis (DC.) Danser	Shrub	jì shēng	WS-MD0130	38.89	Wp	Dry	Oral	Boiled in water and the filtrate is taken orally.	Used to treat rheumatic pain.	Musculoskeletal Disorders
					33.33	Wp	Dry	Oral	Boiled in water and the filtrate is taken orally.	Used to treat lower back and knee pain.	Musculoskeletal Disorders
					27.78	Wp	Dry	Oral	Boiled in water and the filtrate is taken orally.	Used to treat sprains and bruises.	Musculoskeletal Disorders
	Taxillus levinei (Merr.) H.S.Kiu	Shrub	jì shēng	WS-MD0131	41.18	Wp	Dry	Oral	Boiled in water and the filtrate is taken orally.	Used to treat rheumatic pain.	Musculoskeletal Disorders
					35.29	Wp	Dry	Oral	Boiled in water and the filtrate is taken orally.	Used to treat lower back and knee pain.	Musculoskeletal Disorders
					23.53	Wp	Dry	Oral	Boiled in water and the filtrate is taken orally.	Used to treat sprains and bruises.	Musculoskeletal Disorders
Lycopodiaceae	*Lycopodium casuarinoides* Spring	Herb	jìn gǔ fēng, dēng lóng cǎo, mǎ wěi sōng jīn	WS-MD0123	26.32	Wp	Dry, Fresh	Oral, Dermal	Boiled in water and the filtrate is taken orally. For external use, pounded and applied to the affected area.	Used to treat rheumatic joint pain.	Musculoskeletal Disorders
					26.32	Wp	Dry, Fresh	Oral, Dermal	Boiled in water and the filtrate is taken orally. For external use, pounded and applied to the affected area.	Used to treat sprains and bruises.	Musculoskeletal Disorders
					21.05	Wp	Dry, Fresh	Oral	Boiled in water and the filtrate is taken orally.	Used to treat irregular menstruation.	Obstetrics, Gynaecology and Urinary Disorders

	Lycopodium japonicum Thunb.	Herb	shēn jīn cǎo	WS-MD0061	40.00	Wp	Dry, Fresh	Oral, Dermal	Boiled in water and the filtrate is taken orally. For external use, pounded and applied to the affected area.	Used to treat rheumatic joint pain.	Musculoskeletal Disorders
					33.33	Wp	Dry, Fresh	Oral, Dermal	Boiled in water and the filtrate is taken orally. For external use, pounded and applied to the affected area.	Used to treat sprains and bruises.	Musculoskeletal Disorders
					26.67	Wp	Dry, Fresh	Oral	Boiled in water and the filtrate is taken orally.	Used to treat irregular menstruation.	Obstetrics, Gynaecology and Urinary Disorders
Lygodiaceae	*Lygodium circinnatum* (Burm.f.) Sw.	Herb	jīn shā jué, xǐ wǎn téng, sào bǎ téng, hǎi jīn shā	WS-MD0062	62.50	Wp	Dry, Fresh	Oral	Boiled in water and the filtrate is taken orally.	Used to treat urinary tract infections or stones.	Infection/Immune Disorders
					37.50	Wp	Dry, Fresh	Oral	Boiled in water and the filtrate is taken orally.	Used to treat nephritis edema.	Obstetrics, Gynaecology and Urinary Disorders
	Lygodium japonicum (Thunb.) Sw.	Herb	jīn shā jué, xǐ wǎn téng, sào bǎ téng, hǎi jīn shā	WS-MD0063	64.29	Wp	Dry, Fresh	Oral	Boiled in water and the filtrate is taken orally.	Used to treat urinary tract infections or stones.	Infection/Immune Disorders
					35.71	Wp	Dry, Fresh	Oral	Boiled in water and the filtrate is taken orally.	Used to treat nephritis edema.	Obstetrics, Gynaecology and Urinary Disorders
	Lygodium polystachyum Wall. ex T.Moore	Herb	jīn shā jué, xǐ wǎn téng,	WS-MD0064	57.14	Wp	Dry, Fresh	Oral	Boiled in water and the filtrate is taken orally.	Used to treat urinary tract infections or stones.	Infection/Immune Disorders

			sào bǎ téng, hǎi jīn shā		42.86	Wp	Dry, Fresh	Oral	Boiled in water and the filtrate is taken orally.	Used to treat nephritis edema.	Obstetrics, Gynaecology and Urinary Disorders
Malvaceae	*Urena lobata* L.	Shrub	dì táo huā, chī tóu pó, bàn biān yuè	WS-MD0139	15.79	Wp	Dry, Fresh	Oral, Dermal	Taken orally as a decoction; for external use, pounded and applied as a poultice.	Used to treat rheumatic pain and joint pain.	Musculoskeletal Disorders
					15.79	Wp	Dry, Fresh	Oral	Boiled in water and the filtrate is taken orally.	Used to treat colds with fever.	Infection/Immune Disorders
					31.58	Wp	Dry, Fresh	Oral	Boiled in water and the filtrate is taken orally.	Used to treat pharyngitis.	Gastrointestinal Disorders
					36.84	Wp	Dry, Fresh	Dermal	For external use, pounded and applied as a poultice.	Used to treat snake bites.	Poisoning/Toxicology
Melastomataceae	*Melastoma dodecandrum* Lour.	Herb	dì niān	WS-MD0069	100.00	Wp	Dry, Fresh	Oral	Boiled in water and the filtrate is taken orally.	Used to treat colds and fever.	Infection/Immune Disorders
Menispermaceae	*Cyclea hypoglauca* (Schauer) Diels	Climber	jīn xiàn fēng, yín bù huàn, bǎi jiě téng	WS-MD0058	9.38	Rt, St	Dry	Oral	Boiled in water and the filtrate is taken orally.	Used to treat sore throat.	Gastrointestinal Disorders
					9.38	Rt, St	Dry	Oral	Boiled in water and the filtrate is taken orally.	Used to treat hepatitis.	Gastrointestinal Disorders
					12.50	Rt, St	Dry	Oral	Boiled in water and the filtrate is taken orally.	Used to treat stomach pain.	Gastrointestinal Disorders
					12.50	Rt, St	Dry	Oral	Boiled in water and the filtrate is taken orally.	Used to treat high blood pressure.	Blood Disorders
					15.63	Rt, St	Dry	Oral	Boiled in water and the filtrate is taken orally.	Used to treat rheumatic bone pain.	Musculoskeletal Disorders
					18.75	Rt, St	Dry	Oral	Boiled in water and the filtrate is taken orally.	Used to treat snake bites.	Poisoning/Toxicology
					21.88	Rt, St	Dry	Oral	Boiled in water and the filtrate is taken orally.	Used to treat boils and carbuncles.	Infection/Immune Disorders

	Paratinospora sagittata (Oliv.) Wei Wang	Climber	jiǔ niú dǎn, dì kǔ dǎn, jiǔ lián zǐ	WS-MD0076	11.54	Tb	Dry	Oral	Boiled in water and the filtrate is taken orally.	Used to treat stomach pain.	Gastrointestinal Disorders
					11.54	Tb	Dry	Oral	Boiled in water and the filtrate is taken orally.	Used to treat pharyngitis.	Gastrointestinal Disorders
					15.38	Tb	Dry	Oral	Boiled in water and the filtrate is taken orally.	Used to treat pneumonia.	Respiratory Disorders
					19.23	Tb	Dry	Oral	Boiled in water and the filtrate is taken orally.	Used to treat enteritis and diarrhea.	Gastrointestinal Disorders
					19.23	Tb	Dry	Oral	Boiled in water and the filtrate is taken orally.	Used to treat snake bites.	Poisoning/Toxicology
					23.08	Tb	Dry	Oral	Boiled in water and the filtrate is taken orally.	Used to treat carbuncles and boils.	Infection/Immune Disorders
Moraceae	*Broussonetia papyrifera* (L.) L'Hér. ex Vent.	Tree	shā pí shù, féi zhū shù	WS-MD0039	28.57	Sd	Dry	Oral	Boiled in water and the filtrate is taken orally.	Used to treat impotence.	Reproductive Disorders
					35.71	Sd	Dry	Oral	Boiled in water and the filtrate is taken orally.	Used to treat lower back and knee weakness.	Musculoskeletal Disorders
					35.71	Sd	Dry	Oral	Boiled in water and the filtrate is taken orally.	Used to treat dizziness due to kidney deficiency.	Obstetrics, Gynaecology and Urinary Disorders
	Ficus pumila L.	Shrub	zhuī gǔ fēng, wáng bù liú xíng, liáng fěn guǒ	WS-MD0033	18.75	Ft	Dry	Oral	Boiled in water and the filtrate is taken orally.	Used to tonify the kidneys and strengthen essence.	Obstetrics, Gynaecology and Urinary Disorders
					18.75	Ft	Dry	Oral	Boiled in water and the filtrate is taken orally.	Used to promote lactation.	Obstetrics, Gynaecology and Urinary Disorders
					25.00	Lv, St	Dry	Oral	Boiled in water and the filtrate is taken orally.	Used to treat rheumatic pain.	Musculoskeletal Disorders
					37.50	Lv, St	Dry	Oral	Boiled in water and the filtrate is taken orally.	Used to treat traumatic injuries.	Musculoskeletal Disorders

	Ficus simplicissima Lour.	Tree	wǔ zhǎo fēng, wǔ zhǐ máo táo	WS-MD0087	46.67	Rt	Dry	Oral	Boiled in water and the filtrate is taken orally.	Used to treat weakness after illness.	Musculoskeletal Disorders
					26.67	Rt	Dry	Oral	Boiled in water and the filtrate is taken orally.	Used to treat postpartum agalactia.	Obstetrics, Gynaecology and Urinary Disorders
					26.67	Rt	Dry	Oral	Boiled in water and the filtrate is taken orally.	Used to treat cough due to lung deficiency.	Respiratory Disorders
	Maclura cochinchinensis (Lour.) Corner	Tree	chuān pò shí	WS-MD0065	25.00	Rt	Dry	Oral	Taken orally as a decoction or soaked in alcohol.	Used to treat jaundice-type hepatitis.	Gastrointestinal Disorders
					16.67	Rt	Dry	Oral	Taken orally as a decoction or soaked in alcohol.	Used to treat gastric and duodenal ulcers.	Gastrointestinal Disorders
					20.83	Rt	Dry	Oral	Taken orally as a decoction or soaked in alcohol.	Used to treat urinary tract stones.	Obstetrics, Gynaecology and Urinary Disorders
					20.83	Rt	Dry	Oral	Taken orally as a decoction or soaked in alcohol.	Used to treat rheumatic joint pain.	Musculoskeletal Disorders
					16.67	Rt	Dry	Oral	Taken orally as a decoction or soaked in alcohol.	Used to treat sprains and bruises.	Musculoskeletal Disorders
Myrtaceae	*Psidium guajava* L.	Tree	jī shǐ guǒ	WS-MD0156	100.00	Lv	Dry	Oral	Boiled in water and the filtrate is taken orally.	Used to treat stomach pain.	Gastrointestinal Disorders
Nephrolepidaceae	*Nephrolepis cordifolia* (L.) C.Presl	Herb	bīng kāi tái, fèng huáng dàn, shèn jué	WS-MD0075	45.45	Tb	Dry, Fresh	Oral	Boiled in water and the filtrate is taken orally.	Used to treat colds and fever.	Infection/Immune Disorders
					54.55	Tb	Dry, Fresh	Oral	Boiled in water and the filtrate is taken orally.	Used to treat infantile malnutrition.	Blood Disorders

Oleaceae	*Jasminum lanceolaria* Roxb.	Shrub	pò gǔ fēng, sàn gǔ téng, suì gǔ fēng, guāng qīng xiāng téng	WS-MD0109	35.71	Rt, St	Dry, Fresh	Oral, Dermal	Taken orally as a decoction; for external use, pounded and applied as a poultice or decocted in water for washing.	Used to treat rheumatoid arthritis.	Musculoskeletal Disorders
					42.86	Rt, St	Dry, Fresh	Oral, Dermal	Taken orally as a decoction; for external use, pounded and applied as a poultice or decocted in water for washing.	Used to treat sprains and bruises.	Musculoskeletal Disorders
					21.43	Rt, St	Fresh	Dermal	For external use, pounded and applied as a poultice or decocted in water for washing.	Used to treat unidentified boils and carbuncles.	Infection/Immune Disorders
Orobanchaceae	*Striga asiatica* (L.) Kuntze	Herb	ǎi jiǎo zǐ, gān jī cǎo, dú jiǎo jīn	WS-MD0126	40.00	Wp	Dry	Oral	Boiled in water and the filtrate is taken orally.	Used to treat infantile malnutrition.	Blood Disorders
					60.00	Wp	Dry	Oral	Boiled in water and the filtrate is taken orally.	Used to treat infantile diarrhea.	Gastrointestinal Disorders
Phyllanthaceae	*Phyllanthus emblica* L.	Tree	niú gān guǒ, niú gān zǐ	WS-MD0081	46.15	Ft	Fresh	Oral	Taken orally as a decoction or chewed.	Used to treat colds with fever.	Infection/Immune Disorders
					53.85	Ft	Fresh	Oral	Taken orally as a decoction or chewed.	Used to treat sore throat and cough.	Gastrointestinal Disorders
	Phyllanthus eriocarpus (Champ. ex Benth.) Müll.Arg.	Shrub	mǎi bèi xīn pán liàng, qī dà gū, máo qī gōng, qī dà bó	WS-MD0082	14.29	Lv, Rt, St	Dry, Fresh	Oral	Boiled in water and the filtrate is taken orally.	Used to treat acute gastroenteritis.	Gastrointestinal Disorders
					14.29	Lv, Rt, St	Dry, Fresh	Oral	Boiled in water and the filtrate is taken orally.	Used to treat acute sore throat.	Gastrointestinal Disorders
					19.05	Lv, Rt, St	Dry, Fresh	Oral	Boiled in water and the filtrate is taken orally.	Used to treat acute toothache.	Gastrointestinal Disorders

					23.81	Lv, Rt	Fresh	Dermal	For external use, Applied as a decocted in water for washing.	Used to treat acute urticaria.	Skin Disorders
					28.57	Lv, Rt	Fresh	Dermal	For external use, Applied as a decocted in water for washing.	Used to treat acute eczema.	Skin Disorders
	Phyllanthus lucens Poir.	Shrub	hēi miàn yè, hēi miàn shén, guǐ huà fú	WS-MD0083	27.27	Lv, Rt	Dry, Fresh	Oral	Boiled in water and the filtrate is taken orally.	Used to treat colds.	Infection/Immune Disorders
					18.18	Lv, Rt	Dry, Fresh	Oral	Boiled in water and the filtrate is taken orally.	Used to treat bronchitis.	Respiratory Disorders
					18.18	Lv, Rt	Dry, Fresh	Oral	Boiled in water and the filtrate is taken orally.	Used to treat tonsillitis.	Respiratory Disorders
					22.73	Lv, Rt	Dry, Fresh	Oral	Boiled in water and the filtrate is taken orally.	Used to treat acute gastroenteritis.	Gastrointestinal Disorders
					13.64	Lv, Rt	Fresh	Dermal	For external use, Applied as a decocted in water for washing.	Used to treat acute eczema.	Skin Disorders
	Phyllanthus puber (L.) Müll.Arg.	Shrub	jīn gǔ fēng, yě nán guā, dì jīn guā, suàn pán zi	WS-MD0084	29.41	Wp	Dry, Fresh	Oral	Boiled in water and the filtrate is taken orally.	Used to treat dysentery, diarrhea, and enteritis.	Gastrointestinal Disorders
					29.41	Wp	Fresh	Dermal	For external use, pounded and applied to the affected area.	Treatment of snake and centipede bites	Poisoning/Toxicology
					41.18	Wp	Fresh	Dermal	For external use, Applied as a decocted in water for washing.	Used to treat eczema and allergic dermatitis.	Skin Disorders
	Phyllanthus urinaria L.	Herb	yè xià zhū, yè guān mén,	WS-MD0085	21.43	Wp	Dry, Fresh	Oral	Boiled in water and the filtrate is taken orally.	Used to treat red, swollen, and painful eyes.	Eye Disorders

			yè hòu zhū, zhēn zhū cǎo		21.43	Wp	Dry, Fresh	Oral	Boiled in water and the filtrate is taken orally.	Used to treat enteritis, diarrhea, and dysentery.	Gastrointestinal Disorders
					57.14	Wp	Dry, Fresh	Oral	Boiled in water and the filtrate is taken orally.	Used to treat urinary tract infections.	Obstetrics, Gynaecology and Urinary Disorders
Piperaceae	*Piper boehmeriifolium* (Miq.) Wall. ex C.DC.	Shrub	dà cháng fēng, shí bā zhèng, shí tiáo huā	WS-MD0144	9.52	Wp	Dry	Oral	Boiled in water and the filtrate is taken orally.	Used to treat colds.	Infection/Immune Disorders
					14.29	Wp	Dry	Oral	Boiled in water and the filtrate is taken orally.	Used to treat rheumatic pain.	Musculoskeletal Disorders
					19.05	Wp	Dry	Oral	Boiled in water and the filtrate is taken orally.	Used to treat bruises and swelling.	Musculoskeletal Disorders
					28.57	Wp	Dry	Oral	Boiled in water and the filtrate is taken orally.	Used to treat amenorrhea.	Obstetrics, Gynaecology and Urinary Disorders
					28.57	Wp	Dry	Oral	Boiled in water and the filtrate is taken orally.	Used to treat stomach pain due to cold.	Infection/Immune Disorders
	Piper hancei Maxim.	Climber	shāo xiāng fēng, là jiāo jiāng, pá yán xiāng, shān lóu	WS-MD0145	10.00	Wp	Dry	Oral	Boiled in water and the filtrate is taken orally.	Used to treat stomach pain due to cold.	Infection/Immune Disorders
					13.33	Wp	Dry	Oral	Boiled in water and the filtrate is taken orally.	Used to treat colds.	Infection/Immune Disorders
					16.67	Wp	Dry	Oral	Boiled in water and the filtrate is taken orally.	Used to treat rheumatic pain.	Musculoskeletal Disorders
					16.67	Wp	Dry	Oral	Boiled in water and the filtrate is taken orally.	Used to treat bruises and swelling.	Musculoskeletal Disorders

					20.00	Wp	Dry	Oral	Boiled in water and the filtrate is taken orally.	Used to treat amenorrhea.	Obstetrics, Gynaecology and Urinary Disorders
					23.33	Wp	Dry	Oral	Boiled in water and the filtrate is taken orally.	Used to treat stomach pain due to cold.	Infection/Immune Disorders
Plumbaginaceae	*Plumbago zeylanica* L.	Shrub	měng lǎo hǔ, bái huā dān	WS-MD0147	28.00	Wp	Dry	Oral	Taken after prolonged decoction, or stewed with lean pork.	Used for rheumatic bone pain.	Musculoskeletal Disorders
					24.00	Wp	Dry	Oral	Taken after prolonged decoction, or stewed with lean pork.	Used for sprains and strains.	Musculoskeletal Disorders
					20.00	Wp	Dry	Oral	Taken after prolonged decoction, or stewed with lean pork.	Used for chronic hepatitis.	Gastrointestinal Disorders
					16.00	Wp	Dry	Oral	Taken after prolonged decoction, or stewed with lean pork.	Used for cirrhosis.	Gastrointestinal Disorders
					12.00	Wp	Dry	Oral	Taken after prolonged decoction, or stewed with lean pork.	Used for psoriasis.	Skin Disorders
Poaceae	*Imperata cylindrica* (L.) Raeusch.	Grass	bái máo, bái máo gēn, máo cǎo gēn	WS-MD0108	42.86	Rz	Fresh	Oral	Boiled in water and the filtrate is taken orally.	Used to treat colds and cough.	Respiratory Disorders
					35.71	Rz	Fresh	Oral	Boiled in water and the filtrate is taken orally.	Used to treat pharyngitis.	Gastrointestinal Disorders
					21.43	Rz	Fresh	Oral	Boiled in water and the filtrate is taken orally.	Used to treat stomatitis.	Gastrointestinal Disorders
	Lophatherum gracile Brongn.	Grass	shān jī gǔ, zhú yè mài	WS-MD0121	30.43	Wp	Dry	Oral	Boiled in water and the filtrate is taken orally.	Used to treat colds and fever.	Gastrointestinal Disorders

			dōng, dàn zhú yè		21.74	Wp	Dry	Oral	Boiled in water and the filtrate is taken orally.	Used to treat mouth ulcers.	Gastrointestinal Disorders
					26.09	Wp	Dry	Oral	Boiled in water and the filtrate is taken orally.	Used to treat pharyngitis.	Gastrointestinal Disorders
					21.74	Wp	Dry	Oral	Boiled in water and the filtrate is taken orally.	Used to treat urinary tract infections.	Obstetrics, Gynaecology and Urinary Disorders
Polygalaceae	*Polygala chinensis* L.	Herb	zǐ bèi jīn niú, dà jīn niú cǎo, dà jīn bù huàn	WS-MD0148	11.76	Wp	Dry	Oral	Stewed with pork bones or pig's trotters and taken orally.	Used to treat jaundice-type hepatitis.	Gastrointestinal Disorders
					23.53	Wp	Dry	Oral	Stewed with pork bones or pig's trotters and taken orally.	Used to treat bronchitis.	Respiratory Disorders
					29.41	Wp	Dry	Oral	Stewed with pork bones or pig's trotters and taken orally.	Used to treat cough.	Respiratory Disorders
					35.29	Wp	Dry	Oral	Stewed with pork bones or pig's trotters and taken orally.	Used to treat chest pain.	Cardiological Disorders
	Polygala fallax Hemsl.	Tree	huáng huā dào shuǐ lián, huáng huā cān	WS-MD0149	40.00	Rt	Dry, Fresh	Oral	Taken orally as a decoction, or stewed with chicken or lean pork.	Used to treat Qi deficiency.	General Tonic
					32.00	Rt	Dry, Fresh	Oral	Taken orally as a decoction, or stewed with chicken or lean pork.	Used to treat anemia after illness.	Blood Disorders
					28.00	Rt	Dry, Fresh	Oral	Taken orally as a decoction, or stewed with chicken or lean pork.	Used to treat postpartum weakness.	Obstetrics, Gynaecology and Urinary Disorders
	Polygala japonica Houtt.	Herb	guā zǐ jīn, zǐ jīn huā, xiǎo	WS-MD0150	25.00	Wp	Dry	Oral	Boiled in water and the filtrate is taken orally.	Used to treat pharyngitis.	Gastrointestinal Disorders

			yuǎn zhì, jīn bù huàn		37.50	Wp	Fresh	Dermal	For external use, pounded and applied to the affected area.	Used to treat sprains and bruises.	Musculoskeletal Disorders
					37.50	Wp	Fresh	Dermal	For external use, pounded and applied to the affected area.	Used to treat snake bites.	Poisoning/Toxicology
	Salomonia cantoniensis Lour.	Herb	guò shān lóng, zhǎn shé jiàn, yī wǎn pào, xì huáng yào	WS-MD0163	46.67	Wp	Fresh	Dermal	For external use, pounded and applied to the affected area.	Used to treat sprains, bruises, swelling and pain.	Musculoskeletal Disorders
					53.33	Wp	Fresh	Dermal	For external use, pounded and applied to the affected area.	Used to treat rheumatic joint pain.	Musculoskeletal Disorders
	Securidaca inappendiculata Hassk.	Shrub	huáng jiǔ niú, chán yì téng, xuè pí téng	WS-MD0170	42.86	Rt, St	Dry, Fresh	Oral, Dermal	Decoction taken orally; externally applied as an alcohol maceration or as a powder mixed with water.	Used to treat rheumatic bone pain, sprains and bruises.	Musculoskeletal Disorders
					57.14	Rt, St	Dry	Oral	Boiled in water and the filtrate is taken orally.	Used to treat acute gastroenteritis.	Gastrointestinal Disorders
Polygonaceae	*Persicaria capitata* (Buch.-Ham. ex D. Don) H. Gross	Herb	tóu huā liǎo, shí mǎng cǎo, shí là liǎo	WS-MD0078	20.00	Wp	Dry, Fresh	Oral	Boiled in water and the filtrate is taken orally.	Used to treat enteritis and diarrhea.	Gastrointestinal Disorders
					25.00	Wp	Dry, Fresh	Oral	Boiled in water and the filtrate is taken orally.	Used to treat urinary tract infection and urinary tract stones.	Infection/Immune Disorders
					25.00	Wp	Fresh	Dermal	For external use, pounded and applied as a poultice or decocted in water for washing.	Used to treat rheumatic pain.	Musculoskeletal Disorders
					30.00	Wp	Fresh	Dermal	For external use, pounded and applied as a poultice	Used to treat eczema.	Skin Disorders

									or decocted in water for washing.		
	Persicaria filiformis (Thunb.) Nakai	Herb	màn jīng fēng, jiǔ lóng pán, rén zì cǎo	WS-MD0079	15.38	Wp	Dry, Fresh	Oral	Boiled in water and the filtrate is taken orally.	Used to treat stomach pain.	Gastrointestinal Disorders
					26.92	Wp	Dry, Fresh	Oral	Boiled in water and the filtrate is taken orally.	Used to treat rheumatic bone pain.	Musculoskeletal Disorders
					19.23	Wp	Dry, Fresh	Oral	Boiled in water and the filtrate is taken orally.	Used to treat dysentery.	Infection/Immune Disorders
					23.08	Wp	Dry, Fresh	Oral	Boiled in water and the filtrate is taken orally.	Used to treat irregular menstruation.	Obstetrics, Gynaecology and Urinary Disorders
					15.38	Wp	Fresh	Dermal	For external use, pounded and applied to the affected area.	Used to treat snake bites.	Poisoning/Toxicology
	Pleuropterus multiflorus (Thunb.) Turcz. ex Nakai	Climber	shǒu wū, yè jiāo téng, qiān xiàn téng, hé shǒu wū	WS-MD0146	13.64	Lv, St, Tb	Dry	Oral	Boiled in water and the filtrate is taken orally.	Used to treat hemorrhoids.	Gastrointestinal Disorders
					36.36	Lv, St, Tb	Dry	Oral	Boiled in water and the filtrate is taken orally.	Used to treat lower back and knee pain.	Musculoskeletal Disorders
					31.82	Lv, St, Tb	Dry	Oral	Boiled in water and the filtrate is taken orally.	Used to treat muscle and bone pain.	Musculoskeletal Disorders
					18.18	Lv, St	Fresh	Dermal	For external use, Applied as a decocted in water for washing.	Used to treat itchy skin.	Skin Disorders
Polypodiaceae	*Drynaria roosii* Nakaike	Herb	má lóu shuāng, gǔ suì bǔ, mǎ liú jiāng, hóu zi jiāng	WS-MD0022	47.62	Rz	Dry, Fresh	Oral, Dermal	Taken orally as a decoction or soaked in alcohol; for external use, an appropriate amount is pounded and applied to the affected area.	Used to treat rheumatoid arthritis.	Musculoskeletal Disorders

					52.38	Rz	Dry, Fresh	Oral, Dermal	Taken orally as a decoction or soaked in alcohol; for external use, an appropriate amount is pounded and applied to the affected area.	Used to treat injuries from falls and blows.	Musculoskeletal Disorders
	Lepisorus carnosus (Hook.) C.F.Zhao, R.Wei & X.C.Zhang	Climber	guā zǐ lián, bào shù lián, bào shí lián	WS-MD0116	46.67	Wp	Dry	Oral	Boiled in water and the filtrate is taken orally.	Used to treat pulmonary tuberculosis.	Respiratory Disorders
					53.33	Wp	Dry	Oral	Boiled in water and the filtrate is taken orally.	Used to treat sore throat.	Gastrointestinal Disorders
	Pyrrosia lingua (Thunb.) Farw.	Herb	yuán jīn, shí lán, shí jiàn, shí wéi	WS-MD0159	27.27	Wp	Dry	Oral	Boiled in water and the filtrate is taken orally.	Used to treat urinary tract infections.	Infection/Immune Disorders
					36.36	Wp	Dry	Oral	Boiled in water and the filtrate is taken orally.	Used to treat cough.	Respiratory Disorders
					36.36	Wp	Dry	Oral	Boiled in water and the filtrate is taken orally.	Used to treat infantile malnutrition.	Blood Disorders
Primulaceae	*Ardisia crenata* Sims	Shrub	xiǎo láng sǎn, xiǎo luó sǎn, làng sǎn gēn, zhū shā gēn	WS-MD0003	9.52	Rt	Dry	Oral	Boiled in water and the filtrate is taken orally.	Used to treat toothache.	Gastrointestinal Disorders
					14.29	Rt	Dry	Oral	Boiled in water and the filtrate is taken orally.	Used to treat sore throat.	Gastrointestinal Disorders
					23.81	Rt	Dry	Oral	Boiled in water and the filtrate is taken orally.	Used to treat menstrual cramps.	Obstetrics, Gynaecology and Urinary Disorders
					23.81	Rt	Dry	Oral, Dermal	Taken orally as a decoction or soaked in alcohol. For external use: Apply the extract after soaking in	Used to treat rheumatoid arthritis.	Musculoskeletal Disorders

									alcohol to the affected area.		
					28.57	Rt	Dry	Oral, Dermal	Taken orally as a decoction or soaked in alcohol. For external use: Apply the extract after soaking in alcohol to the affected area.	Used to treat sprains and bruises.	Musculoskeletal Disorders
	Ardisia crispa (Thunb.) A.DC.	Shrub	bǎi liǎng jīn, zhú yè fēng, dà luó sǎn	WS-MD0004	27.78	Wp	Dry	Oral	Boiled in water and the filtrate is taken orally.	Used to treat sore throat.	Gastrointestinal Disorders
					33.33	Wp	Dry	Oral, Dermal	Taken orally as a decoction or soaked in alcohol. For external use: Apply the extract after soaking in alcohol to the affected area.	Used to treat bruises.	Musculoskeletal Disorders
					38.89	Wp	Dry	Oral, Dermal	Taken orally as a decoction or soaked in alcohol. For external use: Apply the extract after soaking in alcohol to the affected area.	Used to treat rheumatic pain.	Musculoskeletal Disorders
	Ardisia gigantifolia Stapf	Shrub	xuè fēng, zǒu mǎ tāi, zǒu mǎ fēng	WS-MD0005	37.50	Wp	Dry	Oral, Dermal	Taken orally as a decoction or soaked in alcohol. For external use: Apply the extract after soaking in alcohol to the affected area.	Used to treat rheumatic pain.	Musculoskeletal Disorders

					62.50	Wp	Dry	Oral, Dermal	Taken orally as a decoction or soaked in alcohol. For external use: Apply the extract after soaking in alcohol to the affected area.	Used to treat sprains.	Musculoskeletal Disorders
	Ardisia mamillata Hance	Herb	hóng máo zhān, hǔ shé hóng, hóng zhān cǎo	WS-MD0006	46.67	Wp	Dry	Oral	Boiled in water and the filtrate is taken orally.	Used to treat pulmonary tuberculosis.	Respiratory Disorders
					53.33	Wp	Dry	Oral	Boiled in water and the filtrate is taken orally.	Used to treat menstrual disorders.	Obstetrics, Gynaecology and Urinary Disorders
	Ardisia primulifolia Gardner & Champ.	Herb	pū dì luó sǎn, lián zuò zǐ jīn niú, luò dì jīn niú	WS-MD0007	42.86	Wp	Dry	Oral	Boiled in water and the filtrate is taken orally.	Used to treat pulmonary tuberculosis.	Respiratory Disorders
					57.14	Wp	Dry	Oral	Boiled in water and the filtrate is taken orally.	Used to treat menstrual disorders.	Obstetrics, Gynaecology and Urinary Disorders
Pteridaceae	*Pteris semipinnata* L.	Herb	bàn biān qí, bàn biān jué, bàn biān shū	WS-MD0157	40.00	Wp	Dry, Fresh	Oral	Boiled in water and the filtrate is taken orally.	Used to treat dysentery.	Infection/Immune Disorders
					60.00	Wp	Fresh	Dermal	For external use, pounded and applied to the affected area.	Used to treat external bleeding.	Skin Disorders
Ranunculaceae	*Clematis chinensis* Osbeck	Shrub	wēi líng xiān, hēi jiǔ niú, qīng lóng xū	WS-MD0051	21.43	Rt	Dry	Oral	Boiled in water and the filtrate is taken orally.	Used to treat rheumatic pain.	Musculoskeletal Disorders
					35.71	Rt	Dry	Oral	Boiled in water and the filtrate is taken orally.	Used to treat lumbar muscle strain.	Musculoskeletal Disorders
					42.86	Rt	Dry	Oral	Boiled in water and the filtrate is taken orally.	Used to treat sprains.	Musculoskeletal Disorders

Rosaceae	*Agrimonia pilosa* Ledeb.	Herb	xiān hè cǎo, tuō lì cǎo, lóng yá cǎo	WS-MD0013	55.56	Wp	Dry, Fresh	Oral	Boiled in water and the filtrate is taken orally.	Used to treat hematemesis, hematuria, and hematochezia.	Gastrointestinal Disorders
					44.44	Wp	Dry, Fresh	Oral	Boiled in water and the filtrate is taken orally.	Used to treat gastroenteritis.	Gastrointestinal Disorders
	Eriobotrya japonica (Thunb.) Lindl.	Tree	pí pá	WS-MD0030	100.00	Lv	Dry	Oral	Boiled in water and the filtrate is taken orally.	Used to treat cough.	Respiratory Disorders
	Potentilla sundaica (Blume) W.Theob.	Herb	wǔ pí fēng, shé pào, wǔ zhǎo hǔ	WS-MD0153	26.32	Wp	Dry, Fresh	Oral	Boiled in water and the filtrate is taken orally.	Used to treat colds and fever.	Infection/Immune Disorders
					31.58	Wp	Dry, Fresh	Oral	Boiled in water and the filtrate is taken orally.	Used to treat cough.	Respiratory Disorders
					42.11	Wp	Fresh	Dermal	For external use, pounded and applied to the affected area.	Used to treat snake bites.	Poisoning/Toxicology
	Rosa laevigata Michx.	Shrub	jīn yīng zǐ	WS-MD0160	53.85	Ft	Dry	Oral	Taken orally as a decoction or soaked in alcohol.	Used to treat neurasthenia.	Central Nervous System Disorders
					23.08	Ft	Dry	Oral	Taken orally as a decoction or soaked in alcohol.	Used to treat spermatorrhea.	Reproductive Disorders
					23.08	Ft	Dry	Oral	Taken orally as a decoction or soaked in alcohol.	Used to treat enuresis and frequent urination.	Obstetrics, Gynaecology and Urinary Disorders
	Rubus alceifolius Poir.	Shrub	lǎo hǔ pào, bā yuè pào	WS-MD0161	18.75	Rt	Dry	Oral	Boiled in water and the filtrate is taken orally.	Used to treat hepatitis.	Gastrointestinal Disorders
					18.75	Rt	Dry	Oral	Boiled in water and the filtrate is taken orally.	Used to treat herpes zoster.	Infection/Immune Disorders
					25.00	Rt	Dry	Oral	Boiled in water and the filtrate is taken orally.	Used to treat sprains and bruises.	Musculoskeletal Disorders

					37.50	Rt	Dry	Oral	Boiled in water and the filtrate is taken orally.	Used to treat rheumatic bone pain.	Musculoskeletal Disorders
Rubiaceae	*Dimetia hedyotidea* (DC.) T.C.Hsu	Shrub	bān shā cǎo, bái téng cǎo, niú bái téng	WS-MD0018	12.50	Wp	Dry, Fresh	Oral	Boiled in water and the filtrate is taken orally.	Used to treat colds and fever.	Infection/Immune Disorders
					12.50	Wp	Dry, Fresh	Oral	Boiled in water and the filtrate is taken orally.	Used to treat pneumonia and cough.	Respiratory Disorders
					20.83	Wp	Fresh	Dermal	For external use, pounded and applied as a poultice or decocted in water for washing.	Used to treat rheumatic pain.	Musculoskeletal Disorders
					25.00	Wp	Fresh	Dermal	For external use, pounded and applied as a poultice or decocted in water for washing.	Used to treat itchy skin.	Skin Disorders
					29.17	Wp	Fresh	Dermal	For external use, pounded and applied as a poultice or decocted in water for washing.	Used to treat snake bites.	Poisoning/Toxicology
	Gardenia jasminoides J.Ellis	Shrub	shān zhī zi, huáng zhī zi	WS-MD0092	30.43	Ft	Dry	Oral	Boiled in water and the filtrate is taken orally.	Used to treat colds and fever.	Infection/Immune Disorders
					21.74	Ft	Dry	Oral	Boiled in water and the filtrate is taken orally.	Used to treat jaundice-type hepatitis.	Gastrointestinal Disorders
					26.09	Ft	Dry	Oral	Boiled in water and the filtrate is taken orally.	Used to treat toothache and mouth ulcers.	Gastrointestinal Disorders
					21.74	Ft	Dry	Oral	Boiled in water and the filtrate is taken orally.	Used to treat urethritis.	Obstetrics, Gynaecology and Urinary Disorders
	Mussaenda divaricata Hutch.	Shrub	yù yè jīn huā, tǔ gān cǎo	WS-MD0072	35.71	Wp	Dry	Oral	Boiled in water and the filtrate is taken orally.	Used to treat colds and fever.	Infection/Immune Disorders

				35.71	Wp	Dry	Oral	Boiled in water and the filtrate is taken orally.	Used to treat bronchitis.	Respiratory Disorders
				28.57	Wp	Dry	Oral	Boiled in water and the filtrate is taken orally.	Used to treat rheumatic pain.	Musculoskeletal Disorders
Mussaenda kwangsiensis H.L.Li	Shrub	yù yè jīn huā, tǔ gān cǎo	WS-MD0073	35.71	Wp	Dry	Oral	Boiled in water and the filtrate is taken orally.	Used to treat colds and fever.	Infection/Immune Disorders
				35.71	Wp	Dry	Oral	Boiled in water and the filtrate is taken orally.	Used to treat bronchitis.	Respiratory Disorders
				28.57	Wp	Dry	Oral	Boiled in water and the filtrate is taken orally.	Used to treat rheumatic pain.	Musculoskeletal Disorders
Mussaenda pubescens W.T.Aiton	Shrub	yù yè jīn huā, bái zhǐ sǎn, shān gān cǎo, bái zhǐ shàn	WS-MD0074	35.71	Wp	Dry	Oral	Boiled in water and the filtrate is taken orally.	Used to treat colds and fever.	Infection/Immune Disorders
				35.71	Wp	Dry	Oral	Boiled in water and the filtrate is taken orally.	Used to treat bronchitis.	Respiratory Disorders
				28.57	Wp	Dry	Oral	Boiled in water and the filtrate is taken orally.	Used to treat rheumatic pain.	Musculoskeletal Disorders
Scleromitrion diffusum (Willd.) R. J. Wang	Herb	shé shé cǎo, shé zǒng guǎn, bái huā shé shé cǎo	WS-MD0168	30.43	Wp	Dry, Fresh	Oral	Boiled in water and the filtrate is taken orally.	Used to treat cough and asthma due to lung heat.	Respiratory Disorders
				17.39	Wp	Dry, Fresh	Oral	Boiled in water and the filtrate is taken orally.	Used to treat tonsillitis.	Respiratory Disorders
				17.39	Wp	Dry, Fresh	Oral	Boiled in water and the filtrate is taken orally.	Used to treat pharyngitis.	Gastrointestinal Disorders
				21.74	Wp	Fresh	Dermal	For external use, pounded and applied as a poultice or decocted in water for washing.	Used to treat snake bites.	Poisoning/Toxicology
				13.04	Wp	Dry, Fresh	Oral	Boiled in water and the filtrate is taken orally.	Used to treat tumors.	Cancer

	Uncaria rhynchophylla (Miq.) Miq.	Shrub	shuāng gōu zuàn, gōu téng	WS-MD0138	10.34	St	Dry	Oral	Boiled in water and the filtrate is taken orally.	Used to treat dizziness and headache due to wind-heat.	Central Nervous System Disorders
					10.34	St	Dry	Oral	Boiled in water and the filtrate is taken orally.	Used to treat infantile high fever.	Infection/Immune Disorders
					13.79	St	Dry	Oral	Boiled in water and the filtrate is taken orally.	Used to treat convulsions.	Central Nervous System Disorders
					27.59	St	Dry	Oral	Boiled in water and the filtrate is taken orally.	Used to treat hypertension.	Blood Disorders
					20.69	St	Fresh	Dermal	For external use, Applied as a decocted in water for washing.	Used to treat rheumatoid arthritis.	Musculoskeletal Disorders
					17.24	St	Fresh	Dermal	For external use, Applied as a decocted in water for washing.	Used to treat sprains and bruises.	Musculoskeletal Disorders
Rutaceae	*Tetradium ruticarpum* (A.Juss.) T.G.Hartley	Tree	chá là, yě wú yú, wú zhū yú	WS-MD0132	38.46	Ft	Dry	Oral	Boiled in water and the filtrate is taken orally.	Used to treat abdominal distension and pain.	Gastrointestinal Disorders
					23.08	Ft	Dry	Oral	Boiled in water and the filtrate is taken orally.	Used to treat hernia.	Reproductive Disorders
					38.46	Ft	Dry	Oral	Boiled in water and the filtrate is taken orally.	Used to treat toothache.	Gastrointestinal Disorders
	Zanthoxylum asiaticum (L.) Appelhans, Groppo & J.Wen	Climber	fēi lóng zhǎng xuè	WS-MD0143	28.57	Rt, St	Dry	Oral	Boiled in water and the filtrate is taken orally.	Used to treat rheumatic pain.	Musculoskeletal Disorders
					23.81	Rt, St	Dry	Oral	Boiled in water and the filtrate is taken orally.	Used to treat stomachache.	Gastrointestinal Disorders
					19.05	Rt, St	Fresh	Dermal	For external use, pounded and applied to the affected area.	Used to treat sprains and bruises.	Musculoskeletal Disorders

					14.29	Rt, St	Fresh	Dermal	For external use, pounded and applied to the affected area.	Used to treat bleeding from cuts.	Skin Disorders
					14.29	Rt, St	Dry	Oral	Boiled in water and the filtrate is taken orally.	Used to treat amenorrhea and dysmenorrhea.	Obstetrics, Gynaecology and Urinary Disorders
Sabiaceae	*Sabia fasciculata* Lecomte ex L.Chen	Climber	xiǎo sǎn gǔ fēng, xiǎo fā sàn, qīng fēng téng	WS-MD0162	30.00	Wp	Dry	Oral	Taken orally as a decoction or soaked in alcohol.	Used to treat rheumatic bone pain.	Musculoskeletal Disorders
					25.00	Wp	Dry	Oral	Taken orally as a decoction or soaked in alcohol.	Used to treat nephritis edema.	Obstetrics, Gynaecology and Urinary Disorders
					15.00	Wp	Dry	Oral	Taken orally as a decoction or soaked in alcohol.	Used to treat goiter.	Blood Disorders
					30.00	Wp	Fresh	Dermal	For external use, pounded and applied to the affected area.	Used to treat traumatic injuries.	Skin Disorders
Santalaceae	*Viscum coloratum* (Kom.) Nakai	Shrub	jì shēng	WS-MD0142	29.41	Tb	Dry, Fresh	Oral, Dermal	Taken orally as a decoction; for external use, pounded and applied as a poultice.	Used to treat rheumatic pain.	Musculoskeletal Disorders
					35.29	Tb	Dry, Fresh	Oral, Dermal	Taken orally as a decoction; for external use, pounded and applied as a poultice.	Used to treat lower back pain.	Musculoskeletal Disorders
					35.29	Tb	Dry, Fresh	Oral, Dermal	Taken orally as a decoction; for external use, pounded and applied as a poultice.	Used to treat knee weakness.	Musculoskeletal Disorders
Saururaceae	*Houttuynia cordata* Thunb.	Herb	yú xīng cǎo	WS-MD0102	50.00	Wp	Dry, Fresh	Oral	Boiled in water and the filtrate is taken orally.	Used to treat enteritis.	Gastrointestinal Disorders

					50.00	Wp	Dry, Fresh	Oral	Boiled in water and the filtrate is taken orally.	Used to treat dysentery.	Infection/Immune Disorders
Schisandraceae	*Kadsura coccinea* (Lem.) A.C.Sm.	Climber	dà zuàn, hēi lǎo hǔ	WS-MD0111	29.17	Rt	Dry	Oral	Boiled in water and the filtrate is taken orally.	Used to treat rheumatic bone pain.	Musculoskeletal Disorders
					12.50	Rt	Dry	Oral	Boiled in water and the filtrate is taken orally.	Used to treat stomach pain.	Gastrointestinal Disorders
					20.83	Rt	Dry	Oral	Boiled in water and the filtrate is taken orally.	Used to treat postpartum abdominal pain.	Obstetrics, Gynaecology and Urinary Disorders
					25.00	Rt	Dry	Oral	Boiled in water and the filtrate is taken orally.	Used to treat dysmenorrhea.	Obstetrics, Gynaecology and Urinary Disorders
					12.50	Rt	Dry	Oral	Boiled in water and the filtrate is taken orally.	Used to treat hernia.	Reproductive Disorders
	Schisandra henryi C.B.Clarke	Climber	xiǎo huáng zuàn, wǔ wèi zǐ	WS-MD0167	34.78	St	Dry	Oral	Boiled in water and the filtrate is taken orally.	Used to treat rheumatic bone pain.	Musculoskeletal Disorders
					21.74	St	Dry	Oral	Boiled in water and the filtrate is taken orally.	Used to treat vasculitis.	Cardiological Disorders
					26.09	St	Dry	Oral	Boiled in water and the filtrate is taken orally.	Used to treat bruises and swelling, fractures.	Musculoskeletal Disorders
					17.39	St	Dry	Oral	Boiled in water and the filtrate is taken orally.	Used to treat postpartum abdominal pain.	Obstetrics, Gynaecology and Urinary Disorders
Scrophulariaceae	*Buddleja asiatica* Lour.	Tree	bái bèi fēng, jiān wěi fēng, bái bèi yè	WS-MD0040	14.29	Rt	Dry	Oral	Boiled in water and the filtrate is taken orally.	Used to treat colds and fever.	Infection/Immune Disorders
					14.29	Lv, Rt, St	Fresh	Dermal	For external use, pounded and applied as a poultice or decocted in water for washing.	Used to treat fractures.	Musculoskeletal Disorders

					33.33	Lv, Rt, St	Fresh	Dermal	For external use, pounded and applied as a poultice or decocted in water for washing.	Used to treat sprains and bruises.	Musculoskeletal Disorders
					38.10	Lv, Rt, St	Fresh	Dermal	For external use, pounded and applied as a poultice or decocted in water for washing.	Used to treat rheumatic pain.	Musculoskeletal Disorders
Selaginellaceae	*Selaginella uncinata* (Desv.) Spring	Herb	cuì yǔ cǎo, cuì yún cǎo	WS-MD0171	44.44	Wp	Dry, Fresh	Oral	Boiled in water and the filtrate is taken orally.	Used to treat cough.	Respiratory Disorders
					55.56	Wp	Fresh	Dermal	For external use, an appropriate amount is decocted in water for washing.	Used to treat rheumatic pain.	Musculoskeletal Disorders
Smilacaceae	*Smilax glabra* Roxb.	Climber	tǔ fú líng	WS-MD0174	27.78	Tb	Dry	Oral	Boiled in water and the filtrate is taken orally.	Used to treat diarrhea.	Infection/Immune Disorders
					27.78	Tb	Dry	Oral	Boiled in water and the filtrate is taken orally.	Used to treat irregular menstruation.	Obstetrics, Gynaecology and Urinary Disorders
					44.44	Tb	Dry	Oral	Boiled in water and the filtrate is taken orally.	Used to treat rheumatic pain.	Musculoskeletal Disorders
Solanaceae	*Lycianthes biflora* (Lour.) Bitter	Herb	máo yào, hóng sī xiàn	WS-MD0122	33.33	Wp	Fresh	Oral	Taken orally as a decoction or cooked with eggs.	Used to treat colds and fever.	Infection/Immune Disorders
					66.67	Wp	Fresh	Dermal	For external use, an appropriate amount of fresh leaves is pounded and applied as a poultice.	Used to treat injuries from falls and blows.	Skin Disorders
	Solanum erianthum D. Don	Shrub	tǔ yān yè, yě yān yè, jiǎ yān yè	WS-MD0175	31.25	Wp	Dry, Fresh	Oral	Boiled in water and the filtrate is taken orally.	Used to treat stomach and abdominal pain.	Gastrointestinal Disorders

					31.25	Wp	Fresh	Dermal	For external use, an appropriate amount is decocted in water for washing.	Used to treat rheumatic pain.	Musculoskeletal Disorders
					37.50	Wp	Fresh	Dermal	For external use, an appropriate amount is decocted in water for washing.	Used to treat eczema.	Skin Disorders
	Solanum lyratum Thunb.	Climber	qiān nián bù làn xīn, bái máo téng	WS-MD0124	25.00	Tb	Dry	Oral	Boiled in water and the filtrate is taken orally.	Used to treat acute gastroenteritis.	Gastrointestinal Disorders
					25.00	Tb	Dry	Oral	Boiled in water and the filtrate is taken orally.	Used to treat acute dysentery.	Infection/Immune Disorders
					50.00	Tb	Dry	Oral	Boiled in water and the filtrate is taken orally.	Used to treat acute pharyngitis.	Gastrointestinal Disorders
Talinaceae	*Talinum paniculatum* (Jacq.) Gaertn.	Herb	yè kāi huā, jiǎ rén shēn	WS-MD0129	100.00	Tb	Dry, Fresh	Oral	Taken orally as a decoction or stewed with pork.	Used to treat weakness and consumptive cough after illness or childbirth.	Obstetrics, Gynaecology and Urinary Disorders
Verbenaceae	*Lantana camara* L.	Shrub	wǔ sè méi	WS-MD0114	100.00	Lv, St	Dry, Fresh	Dermal	For external use: decocted in water for washing.	Used to treat itchy skin.	Skin Disorders
	Verbena officinalis L.	Herb	mǎ biān cǎo	WS-MD0140	57.14	Wp	Dry, Fresh	Oral	Boiled in water and the filtrate is taken orally.	Used to treat colds and fever.	Infection/Immune Disorders
					21.43	Wp	Dry, Fresh	Oral	Boiled in water and the filtrate is taken orally.	Used to treat sore throat.	Gastrointestinal Disorders
					21.43	Wp	Dry, Fresh	Oral	Boiled in water and the filtrate is taken orally.	Used to treat swollen gums.	Gastrointestinal Disorders
Viburnaceae		Shrub		WS-MD0164	31.82	Wp	Fresh	Dermal	For external use, pounded and applied as a poultice	Used to treat rheumatic pain.	Musculoskeletal Disorders

									or decocted in water for washing.		
	Sambucus javanica Reinw. ex Blume		hēi jié fēng, zǒu mǎ fēng, jiē gǔ cǎo		13.64	Wp	Dry	Oral	Boiled in water and the filtrate is taken orally.	Used to treat chronic bronchitis.	Respiratory Disorders
					22.73	Wp	Fresh	Dermal	For external use, pounded and applied as a poultice or decocted in water for washing.	Used to treat sprains and bruises.	Musculoskeletal Disorders
					31.82	Wp	Fresh	Dermal	For external use, pounded and applied as a poultice or decocted in water for washing.	Used to treat fractures.	Musculoskeletal Disorders
Violaceae	*Viola inconspicua* Blume	Herb	dì dīng	WS-MD0141	30.00	Wp	Dry, Fresh	Oral	Boiled in water and the filtrate is taken orally.	Used to treat dysentery.	Infection/Immune Disorders
					30.00	Wp	Dry, Fresh	Oral	Boiled in water and the filtrate is taken orally.	Used to treat pharyngitis.	Gastrointestinal Disorders
					40.00	Wp	Dry, Fresh	Oral	Boiled in water and the filtrate is taken orally.	Used to treat red, swollen, and painful eyes.	Eye Disorders
Vitaceae	*Tetrastigma hemsleyanum* Diels & Gilg	Climber	sān yè qīng	WS-MD0134	45.45	Tb	Dry	Oral	Boiled in water and the filtrate is taken orally.	Used to treat rheumatoid arthritis.	Musculoskeletal Disorders
					54.55	Tb	Dry	Oral	Boiled in water and the filtrate is taken orally.	Used to treat tonsillitis.	Respiratory Disorders
	Tetrastigma planicaule (Hook.f.) Gagnep.	Climber	biǎn gǔ fēng, biǎn dān téng	WS-MD0135	18.75	St	Dry	Oral	Taken orally as a decoction or alcohol infusion	Used to treat rheumatic bone pain.	Musculoskeletal Disorders
					25.00	St	Dry	Oral	Taken orally as a decoction or alcohol infusion	Used to treat lumbar muscle strain.	Musculoskeletal Disorders
					25.00	St	Dry	Oral	Taken orally as a decoction or alcohol infusion	Used to treat indigestion.	Gastrointestinal Disorders

					31.25	St	Dry	Oral	Taken orally as a decoction or alcohol infusion	Used to treat diarrhea.	Infection/Immune Disorders
Zingiberaceae	*Alpinia japonica* (Thunb.) Miq.	Herb	shān jiāng, xiǎo fā sǎn	WS-MD0017	15.00	Wp	Dry, Fresh	Oral	Boiled in water and the filtrate is taken orally.	Used to treat rheumatoid arthritis.	Musculoskeletal Disorders
					25.00	Wp	Dry, Fresh	Oral	Boiled in water and the filtrate is taken orally.	Used to treat sprains and bruises.	Musculoskeletal Disorders
					25.00	Wp	Dry, Fresh	Oral	Boiled in water and the filtrate is taken orally.	Used to treat toothache.	Gastrointestinal Disorders
					35.00	Wp	Dry, Fresh	Oral	Boiled in water and the filtrate is taken orally.	Used to treat stomachache.	Gastrointestinal Disorders

Abbreviation. Bb (bulbil), Bk (bark), Bl (bulb), Ft (fruit), Ic (Inflorescence), Lv (leaf), Ps (pith of the stem), Rt (root), Rz (rhizome), Sd (seed), St (stem or shoot), Tb (tuber), Wp (whole plant).
